# Effectiveness, Reach, Uptake, and Feasibility of Digital Health Interventions for Culturally and Linguistically Diverse Populations Living With Prediabetes Across the Lifespan: Systematic Review and Meta-Analysis

**DOI:** 10.2196/70912

**Published:** 2026-02-19

**Authors:** Lisa Whitehead, Min Zhang, Wai Hang Kwok, Diana Arabiat, Amanda Towell Barnard

**Affiliations:** 1 School of Nursing and Midwifery, Edith Cowan University, 270 Joondalup Drive, Joondalup, 6027, Australia, 0061 863042400; 2 JBI – The University of Jordan Health Research Centre, The University of Jordan, Amman, Jordan; 3 The Department of Nursing, University of Otago, Christchurch, New Zealand; 4 JBI Centre for Evidence Informed Nursing and Midwifery, Edith Cowan University, Joondalup, Australia; 5 Epidemiology, Biostatistics and Health Informatics Department, Public Health Institute (PHI), The University of Jordan, Amman, Jordan

**Keywords:** culturally and linguistically diverse, digital health, glycemic control, meta-analysis, prediabetic state, systematic review

## Abstract

**Background:**

Culturally and linguistically diverse (CaLD) populations are at a higher risk of developing prediabetes; however, the effectiveness and implementation of digital health interventions for prediabetes management in this population are not well understood.

**Objective:**

This review aims to evaluate the effectiveness and implementation of digital health interventions (DHIs) versus usual care for glycemic control in CaLD populations living with prediabetes.

**Methods:**

This review aimed to include people of any age living with prediabetes who are from a CaLD background. Experimental and quasi-experimental studies that compare digital health interventions to usual care, waitlist, or active control were eligible. The primary outcome was glycemic control as measured by hemoglobin A_1c_. A comprehensive search was conducted in CINAHL, Cochrane Library, Embase, MEDLINE, 3 trial registers, and gray literature databases, along with reference lists for additional studies. Studies published in English and published since the inception of each database were included. Statistical analyses included meta-analysis, sensitivity analyses, subgroup analyses, meta-regression, and publication bias assessments. The methodological quality was assessed using the JBI critical appraisal tools, and the quality of evidence was evaluated using Grading of Recommendations, Assessment, Development, and Evaluation to create summary of findings tables. Random-effects models with restricted maximum likelihood estimation were employed.

**Results:**

A total of 14 studies involving 5714 adult participants were included. The meta-analysis showed that DHIs were associated with a reduction in hemoglobin A_1c_ (*P*<.001), though evidence certainty was low (mean difference=−0.14, 95% CI −0.24 to ‐0.05). Effects on fasting blood glucose and body weight remain uncertain. Implementation outcomes demonstrated high uptake (>78.8%), engagement (>80%), and intention rates (89.1%) among CaLD populations with prediabetes. Significant heterogeneity was observed in both randomized controlled trials and pre-post studies. Subgroup analyses revealed significant effects at the 6-month follow-up point only for interventions (*P*<.001). Meta-regression identified comorbidity status as the only significant contributor to heterogeneity (*P*=.02). Sensitivity analyses demonstrated robust significant effects (*P*<.001). Publication bias assessment showed mixed results (Begg *P*=.23, Egger *P*=.02), but trim-and-fill analysis confirmed the robustness of the findings with no missing studies. Despite these positive findings, substantial heterogeneity across most outcomes and low-to-very low certainty evidence limit the reliability of these results, warranting cautious interpretation.

**Conclusions:**

DHIs demonstrate potential for improving glycemic control in CaLD populations living with prediabetes. The observed heterogeneity could be attributed to intervention duration, control type, and participants’ comorbidity status. While the findings related to implementation were encouraging, the certainty of the evidence and substantial heterogeneity suggest that DHIs should be used as adjunctive tools with health care provider involvement rather than stand-alone solutions due to low certainty evidence and substantial heterogeneity. Further rigorous research considering contextual, individual, and cultural factors is needed.

## Introduction

### Background

The global burden of prediabetes is substantial and increasing. Prediabetes, also known as impaired glucose tolerance (IGT) and impaired fasting glucose (IFG), increases the risk of developing type 2 diabetes, cardiovascular disease, and stroke [1,2]. Once diagnosed with prediabetes and without intervention, 5%‐10% of the people per year will progress to a diagnosis of type 2 diabetes, and this rate is higher for specific population groups (eg, South and East Asian people and older adults) [3]. Worldwide, the prevalence of IGT and IFG was 9.1% (464 million) and 5.8% (298 million) in 2021, respectively [2]. High-income countries had the highest rates of prediabetes in 2021, and low-income countries are projected to experience a significant rise in prevalence by 2045 [2]. For some individuals living with prediabetes, early and timely treatment, such as lifestyle changes, can effectively prevent or delay the onset of type 2 diabetes [4]. Thus, effective intervention strategies are needed in low-, middle-, and high-income countries to address the diabetes epidemic.

The use of digital health technologies is well established in diabetes management to support patient self-care [5-7]. Digital health, or eHealth, includes a range of approaches, such as mobile health, telehealth and telemedicine, health information technology, wearable devices, and personalized medicine [8]. Digital health interventions (DHIs) are programs that provide information, communication, support, and networks to people to improve their physical and mental health through the use of digital technologies such as smartphones, websites, and text messaging [7,9]. DHIs can help facilitate tailored interventions and improve accessibility for hard-to-reach populations to affect behavior change [10].

Previous reviews have suggested that DHIs, such as digital health coaching, technology-assisted diabetes prevention programs, and digital health–supported lifestyle change programs, are promising strategies to support people living with prediabetes [11-15], type 1 and type 2 diabetes, and their cardiovascular complications [12-19]. Although there is growing evidence to support the use of DHIs among adults with diabetes or prediabetes, further research is needed to establish the efficacy of DHIs in improving prediabetes-related outcomes among diverse groups [11,15,20]. For example, a recent scoping review reported that while DHIs are acceptable for prediabetes self-management, their effectiveness in reducing the risk of type 2 diabetes is still inconclusive [20].

Studies have reported that people from culturally and linguistically diverse (CaLD) backgrounds have a higher risk of diabetes, associated hospitalizations, and mortality but lower uptake and use of DHIs compared to non-CaLD groups [9,21-23]. The term CaLD in this review is defined as “people born in non-English-speaking countries and/or who do not speak English at home” [23]. This category includes racial or ethnic minority groups, immigrants, and refugees [9]. To promote health equity among CaLD groups living with prediabetes, a comprehensive understanding of the effectiveness, reach, uptake, and feasibility of DHIs within CaLD populations is needed.

A systematic search of PROSPERO, MEDLINE, the Cochrane Database of Systematic Reviews, and the Joanna Briggs Institute (JBI) Evidence Synthesis was conducted, and no ongoing or planned systematic reviews on this targeted population were identified. To address this gap, this systematic review aims to synthesize the current evidence on the effectiveness, reach, uptake, and feasibility of DHIs among CaLD living with prediabetes across the lifespan. The outcome reporting is informed by the Reach, Effectiveness, Adoption, Implementation, and Maintenance framework, which offers a systematic approach to evaluate the implementation and translational potential of health interventions [24,25].

### Review Questions

The review questions were as follows: (1) What is the effectiveness of DHIs versus usual care, waitlist, or active comparator on glycemic control in CaLD populations living with prediabetes? (2) How do these interventions compare in terms of their reach, uptake, and feasibility in CaLD populations living with prediabetes?

## Methods

### Overview

This proposed systematic review was conducted following the JBI methodology for systematic reviews of effectiveness and the *Cochrane Handbook for Systematic Reviews of Interventions* to ensure rigorous standards in addressing methodological issues in meta-analyses. We also used the PRISMA (Preferred Reporting Items for Systematic Reviews and Meta-Analyses) 2020 checklist to ensure clear reporting (Checklist 1) [26]. The protocol was registered with PROSPERO (International Prospective Register of Systematic Reviews; CRD42024556292).

### Inclusion Criteria

#### Participants

Studies that included people of any age living with prediabetes from a CaLD background were considered for inclusion in this review without any limitations based on their gender, diagnostic criteria, or duration of the disease. Prediabetes is defined by the presence of IFG, or IGT, or an elevated hemoglobin A_1c_ (HbA_1c_).

We used an evidence-based definition of CaLD study participants, which refers to those who were born in non-English–speaking countries or those whose main language is not English (eg, immigrants and refugees). Indigenous peoples (eg, First Nations and Indigenous peoples in Australia, Canada, the United States, and New Zealand) were not included in this review as they are classified under a separate definition [23]. Thus, this review included CaLD groups that were (1) born in countries where the official language differs from that of their current country of residence or whose language spoken at home is not the official language of the country where they reside or (2) populations that were described in studies as “ethnically or racially diverse” or “ethnic or racial minority” [9].

#### Interventions

This review considered studies evaluating DHIs. A DHI is defined as a discrete technology functionality or capability designed to achieve a specific objective addressing a health system challenge [27]. We categorized the DHIs into 2 groups: the targeted primary user and the stand-alone or integrated interventions. According to the “Classification of Digital Interventions, Services, and Applications in Health” proposed by the World Health Organization, the targeted primary user category included participants or caregivers, health care providers, managers, and data services [27]. The category of stand-alone or integrated interventions includes independent digital components, such as SMS text messaging services, smartphones or tablets, websites, computer-based programs, and videoconferencing. It also encompasses integrated interventions that combine different digital components. There was no limitation in relation to the intensity, frequency, or duration of interventions.

#### Comparators

This review considered studies that compared DHIs to usual care, waitlist, or an active control group. An active control was defined as a specially designed intervention (eg, physical activity, diet counseling, and health education) delivered either face-to-face or through printed materials.

#### Outcomes

This review considered studies that included the outcomes of effectiveness, reach, uptake, or feasibility of DHIs.

##### Evaluation of Intervention Effectiveness

Effectiveness was defined as “the impact of an intervention on important outcomes, including potential negative effects, quality of life, and economic outcomes” [28]. The primary outcome in this review was the impact on HbA1c (%). Secondary outcomes included fasting plasma glucose (FPG), anthropometric indices (eg, body mass index, weight, waist circumference), and patient-reported outcomes.

##### Evaluation of Implementation Outcomes

Implementation outcomes included reach, uptake, engagement, and feasibility based on the Reach, Effectiveness, Adoption, Implementation, and Maintenance framework. Reach was defined as “the absolute number, proportion, and representativeness of individuals who are willing to participate in a given initiative, intervention, or program” [28]. In this review, we operationalized intervention reach using the eligibility percentage, defined as: Intervention Reach=(Number of eligible participants/Total number of individuals invited)×100%.

Uptake (or adoption) was operationalized as the action of taking up or using the intervention or health promotion program components [28]. We considered reach and uptake at the individual participant level. Intervention uptake was captured through the uptake rate, calculated as follows: Uptake Rate=[(Number of individuals who started participating in the intervention/Total number of eligible individuals invited)×100%], as well as through narrative descriptions of participation (eg, the degree of participation in various components of the intervention).

For feasibility, we considered all information on participant satisfaction, acceptance, adherence, retention rates, and user feedback to gain a nuanced understanding of how interventions were received and implemented.

### Types of Studies

Following the Cochrane “Algorithm to decide whether a review should include non-randomized studies of an intervention or not” [29], we included both experimental and quasi-experimental studies, including randomized controlled trials (pilot RCTs, crossover RCTs, cluster RCTs, and prospective RCTs), nonrandomized controlled trials, pre-post studies, and interrupted time-series studies.

### Search Strategy

The search strategy aimed to locate peer-reviewed published studies. This review utilized a 3-step search strategy. An initial search of MEDLINE (EBSCO) was conducted to identify relevant articles on the topic. A full search strategy, including all identified keywords and index terms, was developed with the assistance of a research librarian.

A comprehensive search was then conducted in 4 databases: CINAHL, Cochrane Library, Embase, and MEDLINE; 3 trial register websites, including ClinicalTrials.gov, the Australian New Zealand Clinical Trials Registry, and the International Clinical Trials Registry Platform; as well as 2 gray literature websites, including ProQuest Dissertations & Theses and OpenGrey.EU. The search strategy was adjusted to suit each database and website included in this review (see the full search strategy in Table S1 in Multimedia Appendix 1).

The reference lists of systematic reviews on similar topics were screened for additional studies. Studies published in English since the inception of each database were included.

### Study Selection

Following the search, all identified citations were collated and uploaded into EndNote (version 20.0; Clarivate Analytics), and duplicates were removed. Following a pilot test, titles and abstracts were screened by 2 independent reviewers for assessment against the inclusion criteria for the review (LW, MZ, WHK). Potentially relevant studies were retrieved in full, and their citation details were imported into the JBI System for the Unified Management, Assessment, and Review of Information (JBI SUMARI) (JBI) [26].

Multiple independent reviewers performed the full-text screening for each record according to the inclusion criteria (ATB, LW, MZ, and WHK). The reasons for the exclusion of full-text studies were recorded. Any disagreements that arose between the reviewers at each stage of the selection process were resolved through discussion with a third reviewer (LW).

### Assessment of Methodological Quality

The revised JBI critical appraisal tool for the assessment of risk of bias for RCTs [30] and quasi-experimental studies [31] was used by 2 independent reviewers (LW and MZ) to assess the methodological quality of the included studies. Any disagreements that arose were resolved through discussion with a third reviewer (ATB). Critical appraisal results were reported in a narrative form and presented in a table. All studies, regardless of their methodological quality results, underwent data extraction and synthesis.

### Data Extraction

Two independent reviewers (LW and MZ) used a standardized JBI data extraction tool in JBI SUMARI (JBI) to extract data. The data extracted included information regarding the participants’ characteristics (eg, age, sample size, percentage of female participants, ethnicity), study methods (eg, study design, country, and settings), intervention details (eg, duration, frequency, components, mode of delivery), and outcomes of significance to the review objective (primary and secondary outcomes). Any disagreements that arose during data extraction were resolved by the decision of a third reviewer (DA).

The authors of the studies were contacted to request missing data or clarification, where required. Where a study was reported in multiple publications, the earliest publication was included, and subsequent ones were identified as duplicates.

### Data Synthesis

Data synthesis was conducted through a statistical meta-analysis and narrative synthesis. Statistical analyses were performed using JBI SUMARI and Stata (version 17.0; JBI) by pooling data from the included studies and generating forest plots, funnel plots, and bubble plots. The final postintervention mean differences were used to present the effect size for continuous data (eg, HbA_1c_, weight). Forest plots present mean differences to account for heterogeneity, with corresponding 95% CIs.

The heterogeneity between the studies was evaluated using the *I*² (1%‐100%) statistic and visualized with Galbraith plots. Due to substantial heterogeneity (*I*²>75%) and the inclusion of pre-post studies, random-effects meta-analyses using the restricted maximum likelihood method were used to pool the data. The sources of heterogeneity were explored through meta-regression, sensitivity analyses, and subgroup analyses. Sensitivity analyses were performed using a leave-one-out meta-analysis to test decisions made regarding the effectiveness of interventions versus comparators on HbA_1c_ levels. Subgroup analyses were conducted based on intervention characteristics and types of control. A qualitative evaluation of the heterogeneity of the included studies was also conducted by comparing participant and study characteristics.

Given that our meta-analysis included 10 or more studies, we performed funnel plot asymmetry analysis, including the Egger test and Begg test, to assess publication bias. Although no significant publication bias was detected, given the substantial heterogeneity (*I*²>50%), a trim-and-fill test was performed to assess the robustness of the pooled results and estimate the potential impact of missing studies. Secondary outcomes were meta-analyzed when sufficient data were available. Furthermore, implementation outcomes were narratively synthesized and presented with supporting tables and figures due to the inappropriateness of statistical pooling.

### Assessing Certainty in the Findings

The Grading of Recommendations, Assessment, Development, and Evaluation (GRADE) approach was followed to grade the certainty of evidence [32]. A Summary of Findings (SoF) table was generated using the web-based software GRADEpro GDT/2015 (McMaster University, ON, Canada) to summarize the strength and reliability of the evidence. At least 2 independent reviewers (ATB and MZ) initially undertook this at the primary outcome level. Any disagreements that arose between the reviewers were resolved through discussion with a third reviewer (LW). Where required, the authors of included studies were contacted to request missing or additional data for clarification. The SoF table presents a ranking of the quality of the evidence based on the risk of bias, indirectness, inconsistency, imprecision, and publication bias. The outcomes reported in the SoF table were HbA_1c_ (%), FPG, and body weight.

## Results

### Study Inclusion

Our initial search yielded 1406 records. After removing 205 duplicate records and those marked as ineligible using EndNote, the remaining articles were imported into JBI SUMARI for further screening, as shown in the PRISMA flow diagram. We removed 1041 articles by assessing titles and abstracts, and a total of 138 articles (126 from databases and trial registries and 12 from website and citation searching) were retrieved for full-text review.

**Figure 1.**
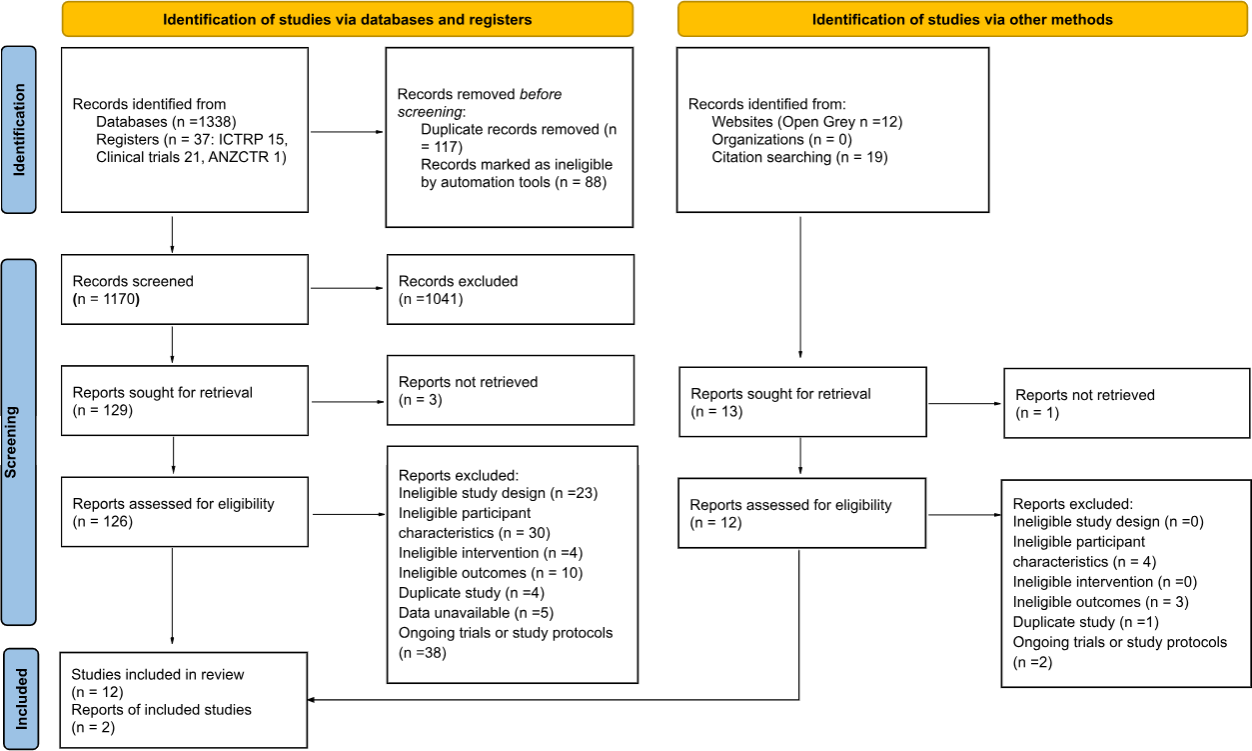
PRISMA (Preferred Reporting Items for Systematic Reviews and Meta-Analyses) flowchart. ANZCTR: Australian New Zealand Clinical Trials Registry; ICTRP: International Clinical Trials Registry Platform.

The search results and the study selection process were reported according to a PRISMA flow diagram in the final review (Figure 1) [33]. Based on the exclusion criteria shown in Figure 1, a total of 14 articles were eligible for inclusion in the systematic review. However, as 1 multinational study reported separate data for each region and another study reported outcomes for 2 distinct intervention completion levels, 17 independent datasets were included in the meta-analysis. The list of excluded studies and their reasons for exclusion is provided in Table S2 in Multimedia Appendix 1.

### Methodological Quality

A total of 14 studies underwent methodological quality assessment. Nine RCTs demonstrated high methodological quality, reporting “Yes” for at least 9 of 13 quality assessment items (Table S3 in Multimedia Appendix 1). All RCTs used intention-to-treat analysis, meaning they included participants in the groups to which they were originally assigned. However, all studies either had unclear results or reported no blinding for participants (0%) and treatment providers (0%). Only 22.22% (2 studies) had outcome assessors who were blind to the treatment assignment. According to Khunti et al. [34], this lack of blinding may be due to the nature of DHIs.

In addition, 5 pre-post studies showed moderate quality, with ≤4 of 9 items rated as unclear or not met (Table S3 in Multimedia Appendix 1). As single-group studies, they inherently could not meet 2 specific items: “There was a control group” (rated as “No”) and “Participants receiving similar treatment/care other than the intervention of interest”(rated as “N/A” due to the lack of between-group comparisons).

### Characteristics of Included Studies

Table S4 in Multimedia Appendix 1 summarizes the characteristics of the included studies. All 14 studies were published between 2014 and 2023 and included a total of 5714 participants. About 9 studies (64.3%) were RCTs, with usual care control (n=4, 28.6%), active comparator (n=1, 7.1%), both usual care and active controls (n=2, 14.3%), and waitlist control (n=2, 14.3%). The remaining 5 studies (35.7%) were pre-post studies.

Over half of the studies were conducted in the United States (n=8), followed by the United Kingdom (n=2). The remaining studies were conducted in the United Kingdom and India, New Zealand, Singapore, and across Sweden, South Africa, and Uganda (n=1 each). The sample size across the 14 studies ranged from 27 to 2390 participants. The mean age of the participants was 53.1 years (SD 10.2), with mean ages across studies ranging from 41.7 to 62.4 years (age range: 18‐88 y). Of the studies that reported the duration of prediabetes (n=4), the duration ranged from 1 to 5 years.

The duration of interventions ranged from 3 to 36 months, with most studies implementing either 6-month or 12-month interventions (n=5 each, 35.7%). Of the 14 included studies, 9 (64.3%) reported follow-up data beyond the endpoint of the intervention, with follow-up periods ranging from 3 to 48 months.

### Primary Effectiveness Outcomes

As Cochrane recommends that randomized trials and nonrandomized studies of interventions should not be combined in a meta-analysis [29], the meta-analyses for RCTs and pre-post studies were conducted separately.

#### Effects of DHIs on HbA_1c_ Levels

**Figure 2.**
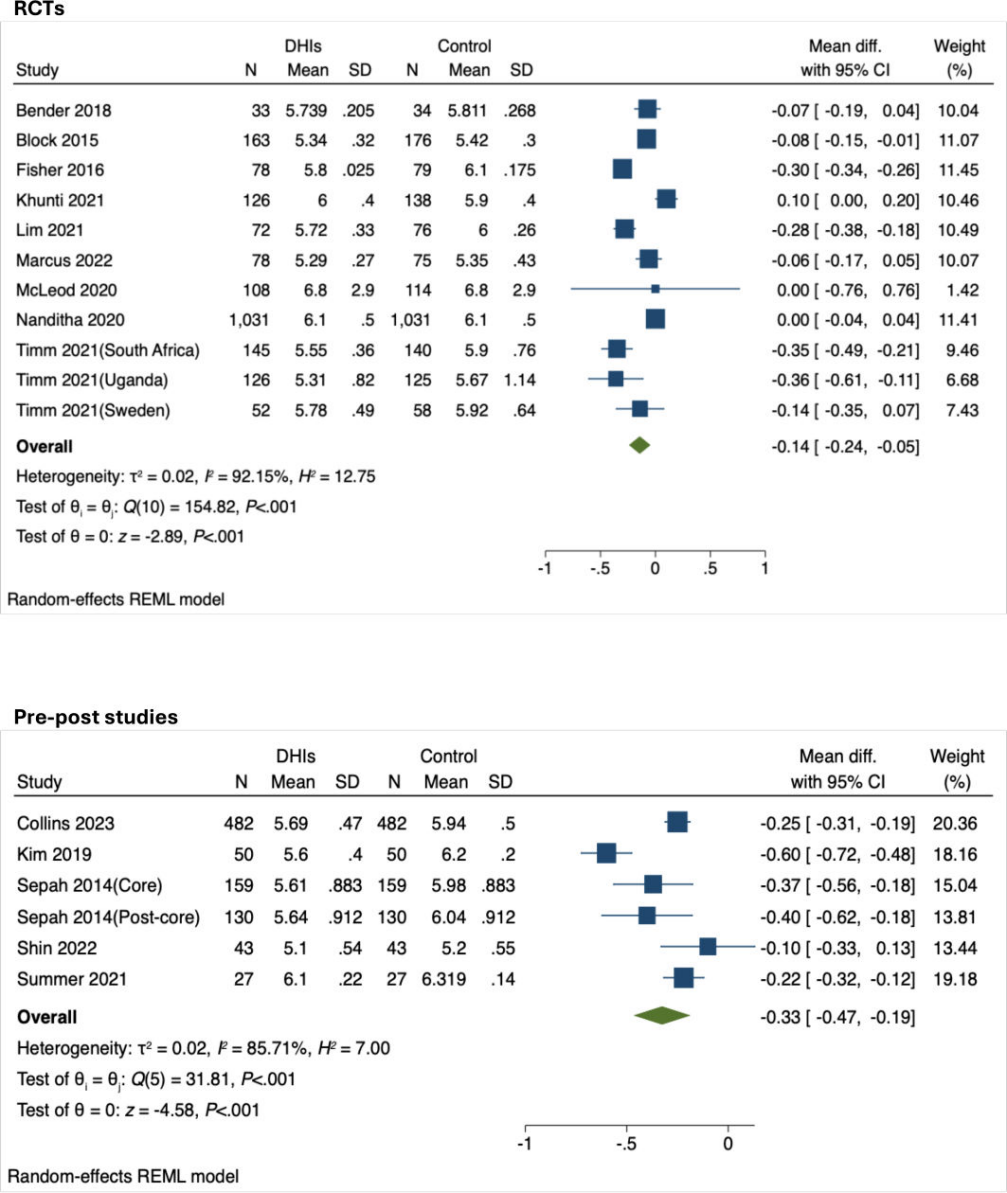
Forest plots of the effect of digital health interventions (DHIs) on hemoglobin A_1c_ (HbA_1c_, %) among culturally and linguistically diverse (CaLD) populations living with prediabetes [34-47]. RCTs: randomized controlled trials; REML: restricted maximum likelihood.

The meta-analysis of RCTs (9 studies with 11 datasets, 4058 participants) showed a small effect on HbA_1c_ reduction (mean difference [MD]=−0.14, 95% CI −0.24 to −0.05, *P*<.001, *I*²=92.15%) favoring DHIs (Figure 2). Pre-post studies (5 studies with 6 datasets, 1782 participants) demonstrated a moderate effect on HbA_1c_ levels post-intervention compared to pre-intervention (MD=−0.33, 95% CI −0.47 to −0.19], *P*<.001, *I*²=85.71%; Figure 2). However, the substantial heterogeneity observed limits the interpretability and generalizability of these findings, warranting cautious interpretation.

#### Sensitivity Analysis

The robustness of the meta-analysis regarding HbA_1c_ levels was examined through a leave-one-out sensitivity analysis, which was produced by excluding a single study to investigate the influence of each study on the overall effect size estimate. The results in Figure S1 in Multimedia Appendix 2 showed that the pooled effect size remained stable (MD ranging from −0.31 to −0.10), with all *P* values <.001, suggesting the robustness of our findings.

At least 2 trials were identified as a potential source of heterogeneity [35,36]. Removing these studies substantially reduced *I²* in both RCTs (92.15%-89.96%) [32] and pre-post studies (85.71%-0.02% [36]; Figure S2 in Multimedia Appendix 2). The statistical significance of pooled effect sizes remained unchanged (all *P*<.001), indicating that while these studies contributed to between-study heterogeneity, they did not alter the significance of the intervention effects.

#### Subgroup Analysis

**Figure 3.**
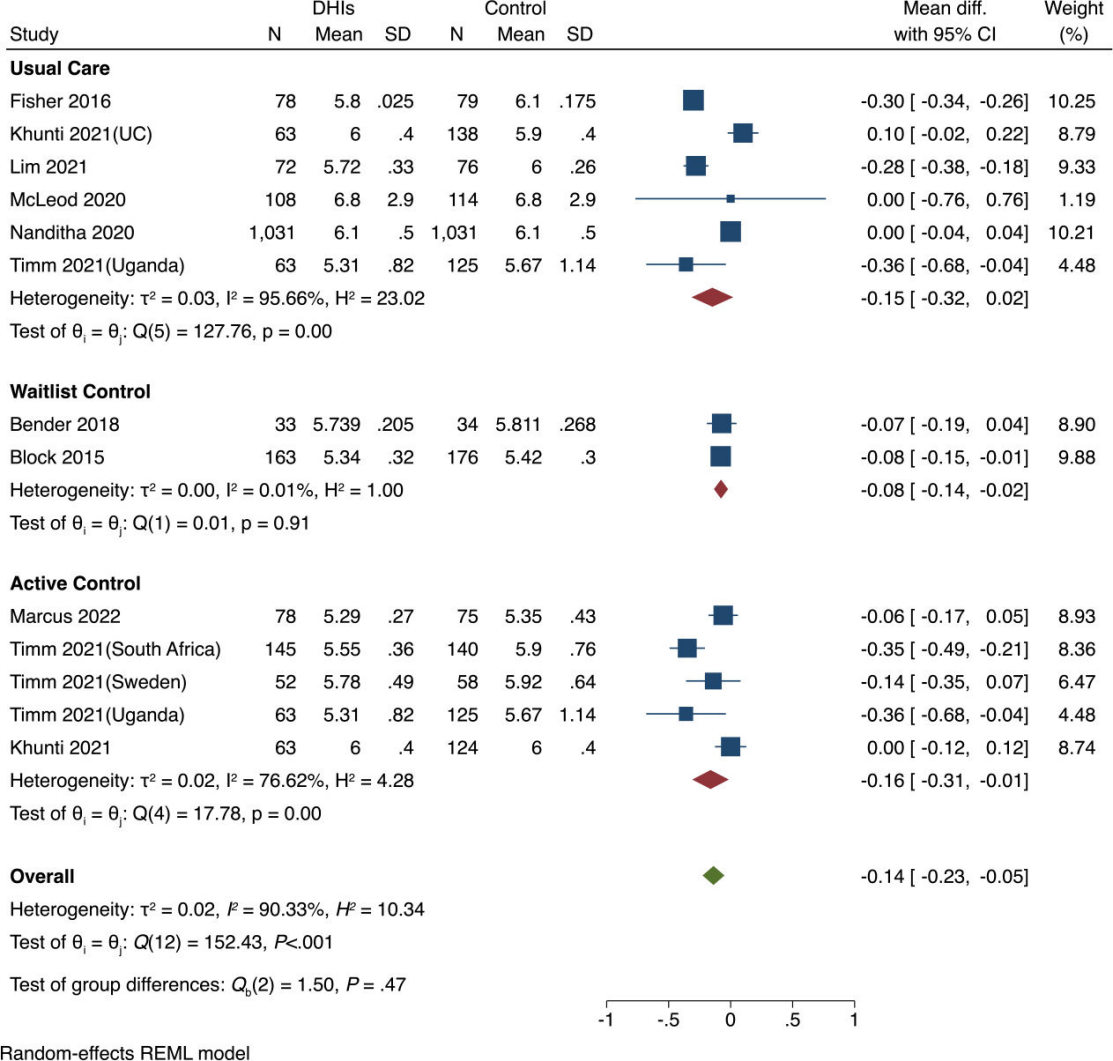
Subgroup analysis of hemoglobin A_1c_ (HbA_1c_, %) by control group types [34,35,37-43]. DHIs: digital health interventions; REML: restricted maximum likelihood.

We conducted subgroup analyses of the included RCTs based on control group type and duration of DHIs (6, 12, or 24 mo), which revealed varying intervention effects across different groups (Figures3  4). Figure 3 indicates that both the usual care and active control subgroups showed significant reductions in HbA_1c_ levels (*P*<.001 for both), while the waitlist control subgroup did not report a significant difference (*P*=.91). Overall, there were no significant differences among the 3 categories of the control subgroups (*P*=.47), indicating that the type of the control group did not significantly influence the effect of DHIs.

**Figure 4.**
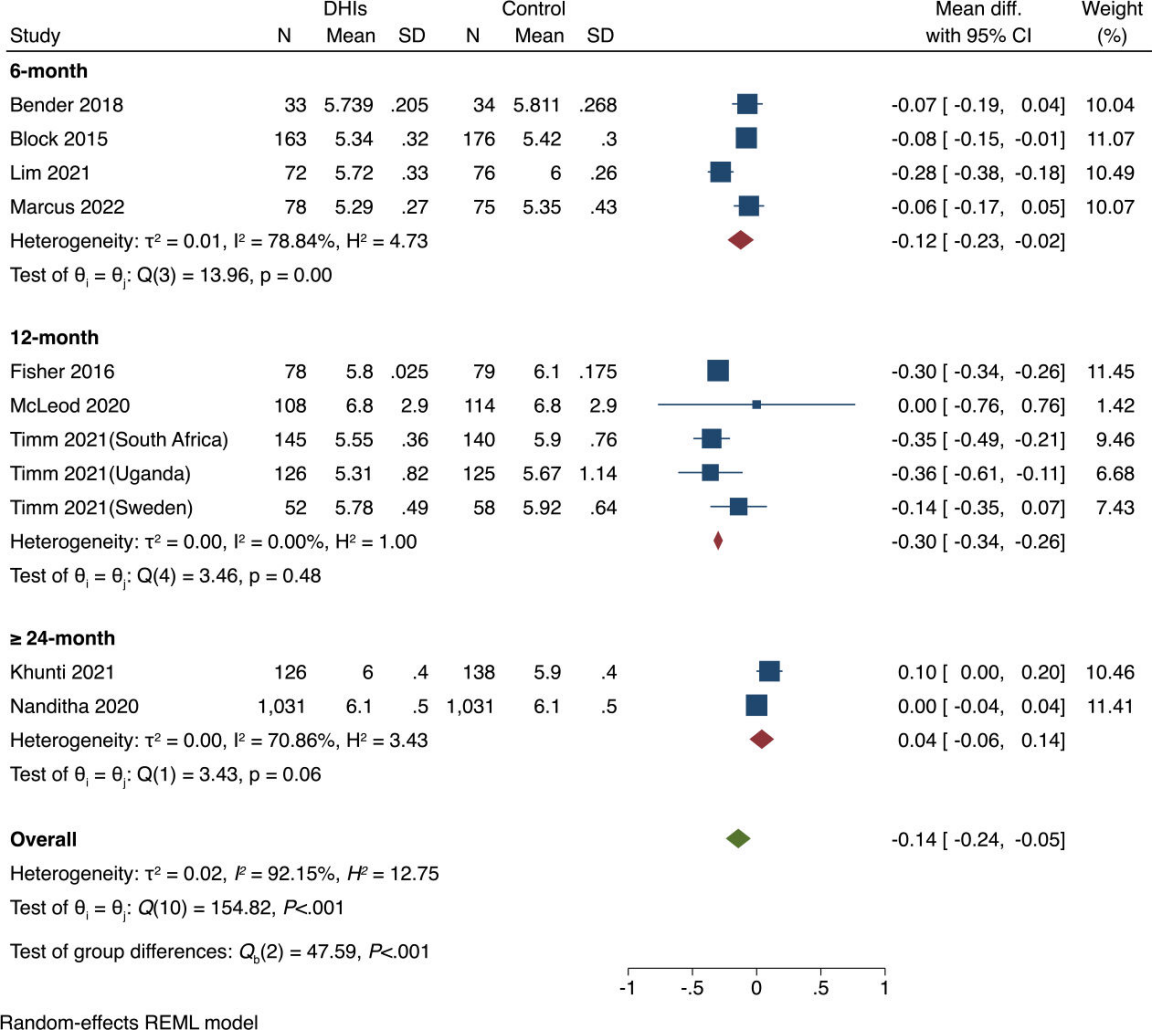
Subgroup analysis of hemoglobin A_1c_ (HbA_1c_, %) by the duration of digital health interventions (DHIs) [34,35,37-43]. REML: restricted maximum likelihood.

In the subgroup analysis of duration of DHIs in Figure 4, only the 6-month intervention group demonstrated a significant reduction in HbA_1c_ (*P*<.001). The test for subgroup differences indicates that the effectiveness of DHIs varies significantly across different intervention durations (*P*<.001). This highlights the importance of careful consideration of intervention duration in the design of DHIs.

Heterogeneity was observed to be low in most subgroups compared to the overall heterogeneity (Figures3  4). For example, the 12-month intervention group showed no heterogeneity (*I*²=0.00%), and the waitlist control group displayed minimal heterogeneity (*I*²=0.01%). The decrease in within-group heterogeneity suggests that the duration of DHIs and control group type may be potential sources of heterogeneity when observed across all studies.

#### Meta-Regression Analysis

**Figure 5.**
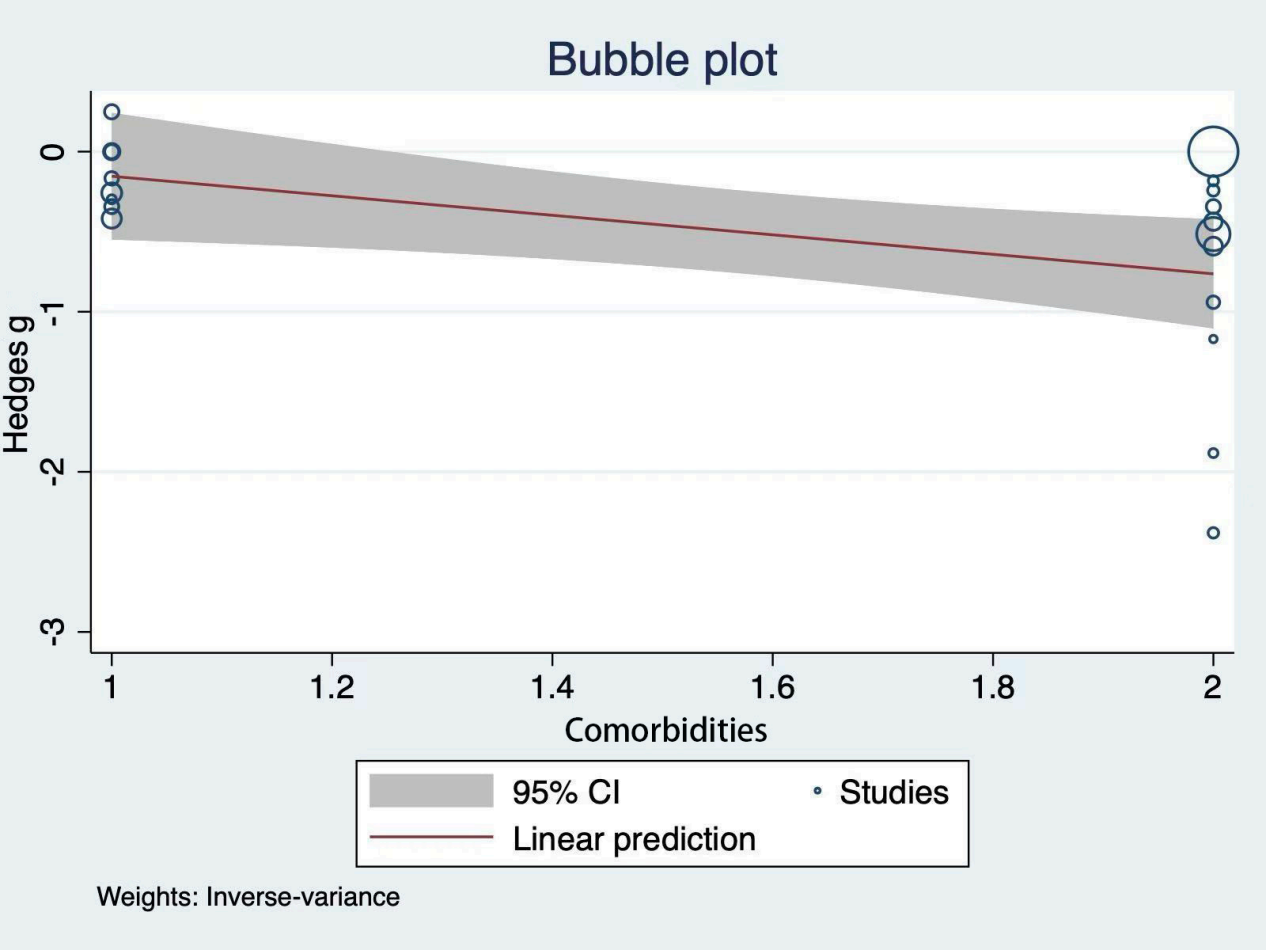
Meta-regression bubble plot showing the relationship between participants' comorbidity status and intervention effects on hemoglobin A_1c_ (HbA_1c_, %).

A meta-regression analysis was performed to explore the sources of heterogeneity for the outcome HbA_1c_ (*I*²=96.23%). Among all covariates tested, including country, sample size, comorbidity status, study design, type of intervention, duration of DHIs, and type of control, only comorbidity status was statistically associated with heterogeneity (*P*=.02), explaining 20.14% of the observed between-study variance (*R*²=20.14%). As shown in the bubble plot in Figure 5, the regression line indicates a negative relationship between comorbidity status and effect size, suggesting that studies reporting participants living with a higher number of comorbidities tend to report smaller intervention effects. However, the wide 95% CI and dispersed study distribution indicate considerable variability in this relationship, limiting the conclusiveness of this association.

#### Publication Bias

Publication bias was assessed using both Begg test and Egger test for HbA_1c_ outcomes. While the Begg test suggested no publication bias (*P*=.23), the Egger test indicated potential publication bias (*P*=.02). We also conducted a trim-and-fill analysis to assess the robustness of our findings, in light of the higher sensitivity of the Egger test and the variety of study designs included.

**Figure 6.**
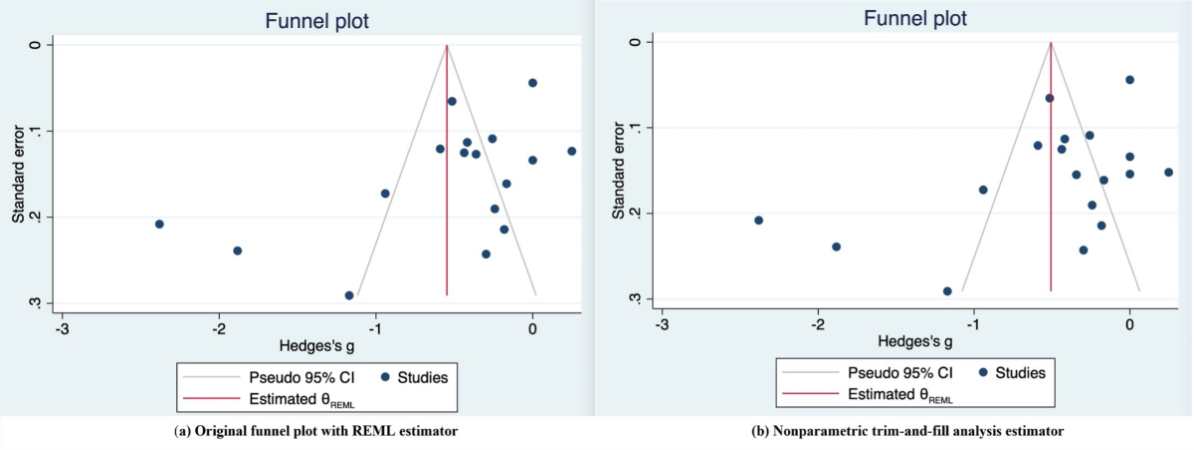
Publication bias assessment using funnel plots. REML: restricted maximum likelihood.

The trim-and-fill analysis identified no missing studies (observed studies=17, imputed studies=0), with the pooled effect size remaining unchanged (95% CI −0.864 to −0.232), suggesting the robustness of the findings. Besides, a visual comparison of the funnel plots in Figure 6 showed that while some asymmetry in study distribution was present, the nonparametric trim-and-fill analysis did not identify any potentially missing studies, as both plots were identical. This suggests that although statistical asymmetry exists, it does not represent true publication bias and therefore does not impact the overall effect estimate.

### Secondary Effectiveness Outcomes

Meta-analyses were conducted for FPG and body weight. RCTs showed improvements favoring DHIs for FPG (4 studies, MD=−0.25, 95% CI −0.55 to 0.04, *P*<.001) and body weight (7 studies, MD=−0.30, 95% CI −0.78 to 0.18, *P*<.001). Pre-post studies demonstrated a reduction in body weight (3 studies with 4 datasets, MD=−0.81, 95% CI −2.16 to −0.53, *P*<.001; Figure S3 in Multimedia Appendix 2). Other secondary outcomes, including body mass index, waist circumference, and self-efficacy, had insufficient data for meta-analysis.

### Implementation Outcomes

#### Reach, Uptake, and Engagement

As shown in Table 1, 9 studies (64.3%) reported intervention reach ranging from 11.9% to 86.6%, and 7 studies reported intervention uptake varying from 78.8% to 100%. Among the 14 included studies, participant engagement was reported in 12 studies, with 5 studies reporting digital platform utilization and 3 studies reporting sustained engagement. Participant engagement varied across interventions, with program completion rates ranging from 66.7% (≥75% core lessons) to 100% (≥40% lessons) and session attendance rates from 80% to 96.5%. Detailed implementation metrics are available in Table S5 in Multimedia Appendix 1.

**Table 1.** Implementation outcomes of digital health interventions.

Author, year	Intervention	Reach (%)	Uptake (%)	Engagement (%)	Retention^a^ (%)
Block et al [37] (2015)	Web-based program	NR^b^	NR	70.8	86.1
Bender et al [38] (2018)	Fit and trim program	NR	78.8	91 (visit attendance)	91
Fischer et al [39] (2016)	Message-augmented intervention	14.6	NR	NR	96.3
Lim et al [40] (2021)	App-based coaching	76.4	100	91.7 (app use)	93.2
Marcus et al [41] (2022)	Enhanced PA^c^	21.4	100	50 (sustained)	62.7
McLeod et al [42] (2020)	BetaMe/Melon digital program	17.6	75.9	74 overall (lower for Māori)	94.4
Nanditha et al [35] (2020)	SMS-based lifestyle support	12.3	NR	NR (2‐3 messages per week)	82
Timm et al [43] (2021)	Telephone-facilitated coaching	2.8‐35.7	NR	NR	49.8‐57.6
Khunti et al [34] (2021)	mHealth^d^ support	11.9	87.4	80 (group attendance)	73.6
Collins et al [44] (2023)	DPP	NR	NR	16.6‐98.4 (across behaviors)	46.9
Kim et al [36] (2019)	Mobile self-help program	79.4	100	96.5 (counseling attendance)	87
Shin et al [45] (2022)	Technology-enhanced PA	NR	NR	93 (device adherence)	100
Summers et al [46] (2021)	Low Carb Program	45	100	66.7 (core lesson)	77.8
Sepah et al [47] (2014)	DPP^e^-based group intervention	86.6	100	65.5 (program completion)	65.5

a Retention rates reflect the final follow-up point for each study.

b NR: not reported.

c PA: physical activity.

d mHealth: mobile health.

e DPP: diabetes prevention program.

#### Feasibility of DHIs

About 13 studies reported outcomes related to feasibility. Retention rates ranged from 46.9% to 100% across the time points (3 mo: 89.1%‐95.9%, 6 mo: 68.6%‐93.2%, 12 mo: 62.7%‐77.8%), while attrition rates varied from 3.7% to 53.1%. Interestingly, program completion was related to participant characteristics (gender, age, education level), with implementation challenges primarily related to language barriers and delivery methods [34,43,47].

#### Cultural Adaptation Strategies

Cultural adaptation strategies were reported in 4 studies, including 2 RCTs (with effect sizes of −0.06 and −0.07, respectively) [38,41] and 2 pre-post studies (with effect sizes of −0.10 and −0.60, respectively; Figure 2) [36,45]. These strategies varied across studies and were grouped into 3 main domains. Linguistic adaptations included native language delivery (Korean, Spanish) [36,41,45]. Content-based adaptations involved culturally adapted psycho-behavioral education addressing unique cultural and motivational factors [36], cultural adaptation of theories (social cognitive theory adapted for Latino culture) [41], and integration of culture-specific food guides and activities [38].

Delivery-mode adaptations included personalized tailoring through individual reports and text messages [36,41], as well as community-engaged approaches, such as small group formats, community health worker involvement, and family-centered methods [36,38,45]. The details of these interventions are provided in Table S6 in Multimedia Appendix 1.

### GRADE Summary of Findings

Based on the SoF in Table 2, DHIs can result in a slight reduction in HbA_1c_ in RCTs (low-certainty evidence) and may reduce HbA1c in pre-post studies (very low-certainty evidence). For secondary outcomes, DHIs may result in little-to-no difference in FPG (MD=−0.25 mmol, 95% CI −0.55 to 0.04; low-certainty evidence) and body weight (MD=−0.3 kg, 95% CI [−0.78, 0.18]; low-certainty evidence) in RCTs. For pre-post studies, DHIs may have little-to-no effect on body weight, but the evidence is very uncertain (MD=−0.81 kg, 95% CI −2.16 to 0.53; very low-certainty evidence). The results of FPG and body weight were derived from Figure S3 in Multimedia Appendix 2.

**Table 2.** Summary of findings: effectiveness of digital health interventions (DHIs) for culturally and linguistically diverse (CaLD) populations living with prediabetes.

Outcomes	Anticipated absolute effects^a^ (95% CI)		Number of participants (studies)	Certainty of the evidence^b^ (GRADE^c^)	Comments
	Risk with control	Risk with DHIs			
HbA_1c^d^_ follow-up: range 3 months to 48 months	The mean HbA_1c_ ranged from 5.35% to 6.8%	MD^e^ 0.14% lower (0.24 lower to 0.05 lower)	4058 (11 RCTs)^f^	⨁⨁◯◯Low^g^^h^	DHIs may result in a slight reduction in HbA_1c_
HbA_1c_ follow-up: range 3 months to 12 months	The mean HbA_1c_ ranged from 5.2% to 6.319%	MD 0.33% lower (0.47 lower to 0.19 lower)	891 (6 nonrandomized studies)	⨁◯◯◯Very low^g^^i^^j^^k^	DHIs may reduce HbA_1c_, but the evidence is very uncertain
FPG^l^ follow-up: range 3 months to 24 months	The mean FPG was 5.07‐6.24 mmol/L	MD 0.25 mmol/L lower (0.55 lower to 0.04 higher)	2616 (4 RCTs)	⨁⨁◯◯Low^g^^m^	DHIs may result in little-to-no difference in FPG
Body weight follow-up: range 6 months to 48 months	The mean body weight ranged from 78.9 to 92.04 kg	MD 0.3 kg lower (0.78 lower to 0.18 higher)	3575 (7 RCTs)	⨁⨁◯◯Low^g^^h^	DHIs may result in little-to-no difference in body weight
Body weight follow-up: range 6 months to 12 months	The mean body weight ranged from 66.9 to 99 kg	MD 0.81 kg lower (2.16 lower to 0.53 higher)	1242 (5 nonrandomized studies)	⨁◯◯◯Very low^g^^h^^i^^n^	DHIs may have little-to-no effect on body weight, but the evidence is very uncertain

a The risk in the intervention group (and its 95% CI) is based on the assumed risk in the comparison group and the relative effect of the intervention (and its 95% CI).

b GRADE Working Group grades of evidence High certainty: we are very confident that the true effect lies close to that of the estimate of the effect. Moderate certainty: we are moderately confident in the effect estimate: the true effect is likely to be close to the estimate of the effect, but there is a possibility that it is substantially different. Low certainty: our confidence in the effect estimate is limited: the true effect may be substantially different from the estimate of the effect. Very low certainty: we have very little confidence in the effect estimate: the true effect is likely to be substantially different from the estimate of effect.

c GRADE: Grading of Recommendations, Assessment, Development, and Evaluation.

d HbA_1c_: hemoglobin A_1c_.

e MD: mean difference.

f RCTs: randomized controlled trials.

g Downgraded 1 level for inconsistency due to substantial statistical heterogeneity (*I*²>80%, *P*<.001), although CIs showed overlap across studies.

h The evidence was downgraded 1 level for imprecision due to wide CIs spanning both benefit and harm.

i Downgraded 1 level for study limitations due to methodological concerns identified by the JBI critical appraisal tool, particularly regarding insufficient follow-up time (0% compliance) and inadequate reporting of loss to follow-up (20% compliance), although other methodological aspects were well addressed.

j Despite being pre-post studies, the evidence directly addressed our research question in terms of population, intervention, and outcomes. Therefore, we did not downgrade for indirectness.

k Evidence was downgraded 1 level for imprecision due to 1 study (16.17% weight) having CIs crossing the null line, which impacts the certainty of the effect estimate.

l FPG: fasting plasma glucose.

m Evidence was downgraded 1 level for imprecision due to CIs crossing the null line in 2 of the 4 studies, despite an adequate total sample size.

n Downgraded 2 levels for very serious inconsistency due to substantial statistical heterogeneity (*I*²>90%, *P*<.001) and lack of overlap in CIs across most studies.

## Discussion

### Principal Findings

This systematic review and meta-analysis examined 14 studies involving 17 articles on the effectiveness, reach, uptake, and feasibility of single- or multi-component DHIs for 5714 participants from CaLD backgrounds living with prediabetes. The meta-analysis indicated that DHIs were effective in improving glycemic control compared to usual care or active controls in both RCTs and pre-post studies (*P*<.001). However, the evidence from the SoF tables indicates low-to-very low certainty, suggesting that while DHIs may slightly improve HbA_1c_, the true effect may differ significantly. Similarly, the effects on FPG and body weight are uncertain. In light of the low-to-very low certainty evidence, DHIs are best used as adjunctive tools in prediabetes management with health care provider involvement, rather than as stand-alone solutions, to maximize effectiveness [6,48]. Regarding intervention implementation, the included studies demonstrated high uptake and strong engagement with DHIs among CaLD populations living with prediabetes, along with satisfactory completion and attendance rates.

### Findings in Context

Our meta-analysis found that DHIs, whether stand-alone or integrated, are effective compared to usual care or active control in managing glycemic control in prediabetes. The findings are congruent with previous reviews [20,49], recently published and focused on all populations, although the consistent results are partly due to the overlap of the included studies. They included 3 studies on HbA1c outcomes, 2 of which were also included in our review. We excluded the other study because it was not related to CaLD populations [50]. This situation potentially reflects the limited evidence, particularly from RCTs, on the effectiveness of DHIs in prediabetes management. Our review of 14 studies involving 11 datasets of RCTs provides more comprehensive and systematic evidence, while also extending the evidence specifically to CaLD populations with prediabetes. It should be noted that the inclusion of 5 pre-post studies resulted in very low certainty evidence for both HbA_1c_ and body weight outcomes, primarily due to inherent design limitations such as temporal confounding and lack of control groups.

Several aspects affect the effectiveness of DHIs in managing blood glucose among CaLD populations living with prediabetes. Our subgroup analysis findings suggest that the duration of intervention should be carefully considered when designing DHIs. The results showed that only 6-month interventions had a significant reduction in HbA_1c_ levels (Figure 4). This pattern aligns with previous evidence showing that intervention effects may be difficult to sustain over time. The Finnish Diabetes Prevention Study demonstrated significant improvements in HbA_1c_ at year 1, with benefits diminishing after year 2 [51]. Similarly, mobile health interventions more generally showed that the impact decreased over time, especially after a 6-month follow-up [52]. One possible explanation for this pattern is the decrease in the retention rates and engagement with DHIs across the time points. Participant retention rates reported across 13 studies showed a notable decline, decreasing from high levels at 3 months to moderate levels at 12 months. Engagement data showed similar temporal patterns; for example, Collins et al [44] noted a drop from 74.0% to 46.9%. Additionally, attrition rates showed greater variability in 12-month studies (range: 3.7%‐53.1%) compared to 6-month studies (range: 6.9%‐15.2%; Table S5 in Multimedia Appendix 1).

Moreover, the quality of implementation and cultural adaptation may explain the variations in the effectiveness observed. The outcomes reported at the 12-month follow-up showed lower consistency in effects compared to the 6-month follow-up data, with nonsignificant pooled results due to 2 studies finding null effects (Figure 4) [42,43]. Timm et al. [43] found that within a multicountry intervention, the Sweden subgroup showed no effect due to language barriers and inconsistent delivery of the intervention, while significant effects were noted in the South African and Ugandan subgroups. Additionally, McLeod et al. [42] observed poor engagement among Māori participants, attributing this challenge to the insufficient involvement of Māori and Pacific populations in the design phases. These findings suggest that support for participants to continue to engage with the elements of the intervention is required. In this context, the follow-up at 6 months indicated an optimal balance between sustained engagement and effectiveness. Only 2 studies examined the impact of interventions at 24 months or longer, indicating the need to further understand the longer-term impact of interventions and how participants can be supported to enhance engagement over the longer term.

The meta-regression data indicated a significant association between comorbidity status and the effectiveness of DHIs. This finding aligns with the Look AHEAD trial, which shows that multimorbidity can reduce the effectiveness of lifestyle interventions for diabetes by creating competing treatment demands and increasing the complexity of self-care [53,54]. However, our findings should be interpreted with caution due to the limited explanatory power (*R*²=20.14%, *P*=.02) and require validation in larger studies before informing the design of interventions. Future research should focus on identifying effect moderators and developing individualized, patient-centered DHIs that incorporate disease monitoring and comorbidity management for those living with multiple comorbidities (eg, cardiovascular disease, hyperlipidemia, arthritis) rather than a “one-size-fits-all” approach. The control of confounding factors is essential in intervention studies. Only one of the included studies reported contamination, where both the intervention and control groups received similar additional treatments (eg, diabetes prevention program classes and weight loss programs) [39]. The consideration and reporting of confounding factors need to be addressed when designing future DHIs.

Implementation outcomes for DHIs among CaLD populations living with prediabetes are encouraging, despite high heterogeneity (Table S5 in Multimedia Appendix 1). Qualitative data from 2 RCTs identified key challenges, including language barriers and delivery methods, in implementing DHIs and emphasized the need to tailor interventions [34,43]. These findings are consistent with those from a previous qualitative systematic review, which emphasized the necessity to incorporate cultural and linguistic perspectives into the design and delivery of DHIs to enhance acceptability, appropriateness, and accessibility for underserved populations [9]. Despite this recognized importance, studies employing cultural adaptation strategies remain limited.

In our review, we identified only 4 studies that integrated cultural adaptations into DHIs, specifically involving Korean Americans, Filipino Americans, and Spanish-speaking Latin Americans [36,38,41,45]. Cultural adaptation in this context refers to comprehensive modifications in content and delivery methods, rather than merely translating language. One study, for example, tailored its DHI for Filipino Americans by incorporating cultural considerations into a mobile- and social media–based program. This included, but was not limited to, how to make healthier Filipino meals, providing a Filipino food guide, and promoting indoor and outdoor activities that reflect Filipino culture [38]. Our findings indicated that studies varied in both the use of cultural adaptation and the specific strategies employed, which may contribute to the observed heterogeneity in HbA_1c_ outcomes. Although subgroup analyses were not feasible with only 2 RCTs employing diverse culturally adapted approaches, the identified strategies provide potential directions for a future intervention design. Evidence supports a tailored approach to the development of information, resources, and interventions for CaLD populations to leverage community resources and expertise and ensure interventions are accessible and relevant to CaLD communities [55]. Researchers and clinicians need to be aware of the importance of creating information, resources, and interventions that are both accessible in terms of language and comprehension and are culturally relevant. Cultural adaptation requires a multilevel approach that simultaneously addresses language, social structures, and practical cultural elements.

### Implications for Future Research

Several knowledge gaps need to be addressed in future research. More rigorous research is needed not only to validate the effectiveness of DHIs but also to understand how DHIs can be optimally designed and implemented across diverse populations [37,39,40]. While our review found that most studies (n=13, 92.9%) employed integrated digital approaches, there is a need for future research to consider person-centered and culturally tailored content in addition to focusing on the impact of digital delivery modes. Further research should examine how contextual factors such as gender, age, and comorbidity influence outcomes [34,35]. Additionally, more research is needed to understand how various cultural adaptation strategies impact the effectiveness and implementation of interventions. This will help identify which components are most effective for managing prediabetes.

There is also a critical need to optimize participant engagement strategies, including recruitment methods, duration of DHIs, and follow-up periods, to improve recruitment, completion, and adherence and further enhance the reliability of research findings. Notably, although this review aimed to include people of all ages with prediabetes, all the included studies focused on adults (≥18 y), highlighting a research gap in interventions designed for children and young people living with prediabetes. Future research should prioritize the development and evaluation of culturally adapted DHIs across the entire lifespan, particularly for pediatric and adolescent populations, where prediabetes prevalence has reached alarming levels globally and intensive lifestyle modification is critically needed [56].

### Strengths

The strengths of this review included the use of rigorous systematic review and meta-analysis guidelines, separate meta-analyses for RCTs and pre-post studies, the restricted maximum likelihood method for robust random-effects analyses, comprehensive heterogeneity exploration through subgroup analyses and meta-regression, leave-one-out sensitivity analyses to assess robustness, a trim-and-fill method for publication bias to ensure reliability, the inclusion of both effectiveness and implementation metrics, and a SoF table to assess the certainty of evidence.

### Limitations

Our review also has several limitations. The high heterogeneity (*I*²>75%) and predominantly low-to-very low GRADE certainty substantially limit the interpretability of our findings, which should be considered preliminary and require validation through larger, higher-quality studies. Lack of blinding in the included RCTs may have introduced performance bias. However, the objective nature of our effectiveness outcomes (eg, HbA_1c_, FPG) and implementation outcomes (eg, retention rates) helps limit measurement bias. Despite our comprehensive, age-inclusive search strategy, no eligible studies were identified targeting children and adolescents from CALD backgrounds, revealing a critical evidence gap in this field. Additionally, most studies were conducted in high-income countries, limiting generalizability to low-resource settings where CaLD populations may face different barriers to health care access and technology adoption. Finally, HbA_1c_ endpoint data from the Swedish arm of a multicenter RCT [43] were not directly reported in the published article. We obtained the original data file (STATA format) from the clinical trial registry and contacted the corresponding author for verification. Means and standard deviations from the original data were calculated for meta-analysis, following Cochrane guidelines on using the available and reliable original data. The results should be interpreted with caution.

### Conclusions

This systematic review and meta-analysis suggests that DHIs may be effective in improving glycemic control; however, the low certainty of the evidence indicates that there may be variations in the true effects, and the impacts on FPG and body weight remain uncertain. The observed heterogeneity may be attributed to differences in duration of DHIs, control type, and comorbidity status, with significant effects observed at the 6-month follow-up time point. Trim-and-fill analysis for publication bias indicated no missing studies and unchanged effect estimates. Implementation metrics showed promising results, with high uptake and engagement among CaLD populations with prediabetes. Future research employing rigorous RCT designs, consideration of contextual factors in intervention design and implementation, and the prioritization of person-centered, culturally tailored content for diverse populations across different geographical locations is needed to enhance the evidence base.

## Supplementary material

10.2196/70912 Multimedia Appendix 1 Search strategy; studies excluded at full-text review; quality assessment; characteristics of included studies; reach, uptake, engagement, and feasibility outcomes of digital health interventions across studies; and summary of cultural adaptation strategies used in digital health interventions.

10.2196/70912 Multimedia Appendix 2 Leave-one-out sensitivity analyses of HbA1c (%) showing the influence of each study on the pooled effect estimates; Forest plots after removing influential studies; and meta-analysis.

10.2196/70912 Checklist 1 PRISMA 2020 checklist.

## Abbreviations

CaLD: culturally and linguistically diverse
DHIs: digital health interventions
FPG: fasting plasma glucose
GRADE: Grading of Recommendations, Assessment, Development, and Evaluation
HbA_1c_: hemoglobin A_1c_
IGT: impaired glucose tolerance
JBI: Joanna Briggs Institute
PRISMA: Preferred Reporting Items for Systematic Reviews and Meta-Analyses
PROSPERO: International Prospective Register of Systematic Reviews
RCT: randomized controlled trial
SoF: Summary of Findings
