# Supplementary material for: Effectiveness, Reach, Uptake, and Feasibility of Digital Health Interventions for Culturally and Linguistically Diverse Populations Living With Prediabetes Across the Lifespan: Systematic Review and Meta-Analysis
Source: JMIR Diabetes. 2026 Feb 19;11:e70912. doi: 10.2196/70912 (PMC12919907; doi:10.2196/70912)
Supplement: Multimedia Appendix 1 [file diabetes-v11-e70912-s001.docx]

## **Multimedia Appendix 1**

## Table S1. Search strategy

**1. MEDLINE (EBSCOhost)**

The search was conducted on 31st May 2024.

| **Search** | **Query** | **Records retrieved** |
| --- | --- | --- |
| S1 | “pre-diabet*” OR prediab* OR MH “Prediabetic State” | 22,732 |
| S2 | TX ( “Culturally and linguistically diverse” ) OR TX “Culturally diverse” OR TX “Linguistically diverse” OR TX “Low English” OR TX Ethnic* OR TX Migrant* OR TX Immigrant* OR TX Emigrant* OR TX “Language barrier” OR TX “Non-english” | 347,983 |
| S3 | MH “Cultural diversity” OR MH ( “Transients and migrants” ) OR MH ( “Emigrants and Immigrants+” ) OR MH “Ethnicity” OR MH “Limited English Proficiency” OR MH “Communication Barriers” OR MH ( “Ethnic and Racial Minorities” ) | 121,464 |
| S4 | S2 OR S3 | 361,933 |
| S5 | TX mhealth OR TX “m-health” OR TX “mobile health” OR TX “mobile app*” OR TX ehealth OR TX “e-health” OR TX Internet OR TX “online health” OR TX “digital health” OR TX “digital intervention*” OR TX tele-medicine OR TX telemedicine | 350,417 |
| S6 | TX telehealth OR TX telecare OR TX “health technolog*” OR MH “Digital Health” OR TX “digital app*” OR MH “Telemedicine” OR TX “smartwatch” OR TX “electronic health” | 172,605 |
| S7 | S5 OR S6 | 452,696 |
| S8 | S1 AND S4 AND S7 | 207 |
| Limiters - English Language  Search modes - Boolean/Phrase | |  |

**2. CINAHL (EBSCOhost CINAHL Plus with Full Text)**

The search was conducted on 26^th^ May 2024.

| **Search** | **Query** | **Records retrieved** |
| --- | --- | --- |
| S1 | TX “pre-diabet*” OR TX prediab* OR TX “pre diab*” OR MH “Prediabetic State” | 18,095 |
| S2 | TX ( “Culturally and linguistically diverse” ) OR TX “Non-english” OR TX “Culturally diverse” OR TX “Linguistically diverse” OR TX “Low English” OR TX Ethnic* OR TX Migrant* OR TX Immigrant* OR TX Emigrant* OR TX “Language barrier” | 395,745 |
| S3 | MH “Cultural diversity” OR MH ( “Transients and migrants” ) OR MH ( “Emigrants and Immigrants+” ) OR MH “Ethnicity” OR MH “Limited English Proficiency” OR MH “Communication Barriers” OR MH ( “Ethnic and Racial Minorities” ) | 23,009 |
| S4 | S2 OR S3 | 408,467 |
| S5 | TX mHealth OR TX “m-health” OR TX “mobile health” OR TX “mobile app*” OR TX ehealth OR TX “e-health” OR TX Internet OR TX “online health” OR TX “digital health” OR TX “digital intervention*” OR TX tele-medicine OR TX telemedicine | 356,723 |
| S6 | TX telehealth OR TX telecare OR TX “health technolog*” OR TX “digital app*” OR MH “Digital Health” OR MH “Telemedicine” OR TX “smartwatch” OR TX “electronic health” | 159,412 |
| S7 | S5 OR S6 | 459,798 |
| S8 | S1 AND S4 AND S7 | **994** |
| Limiters - English Language  Search modes - Boolean/Phrase | |  |

**3. Cochrane Library**

The search was conducted on 26^th^ May 2024.

| **Search** | **Query** | **Records retrieved** |
| --- | --- | --- |
| #1 | (“pre-diabet*”) OR (prediab*) OR (“pre diab*”) OR (“Prediabetic State”):kw | 4,164 |
| #2 | (“Culturally and linguistically diverse”) OR (“Non-english”) OR (“Culturally diverse”) OR (“Linguistically diverse”) OR (“Low English”) | 509 |
| #3 | (Ethnic*) OR (Migrant*) OR (Immigrant*) OR (Emigrant*) OR (“Language barrier”) | 18,163 |
| #4 | (“Cultural diversity”):kw OR (“Transients and migrants”):kw OR (“Emigrants and Immigrants”):kw OR (“Ethnicity”):kw OR (“Limited English Proficiency”):kw | 6,956 |
| #5 | (“Communication Barriers”):kw OR (“Ethnic and Racial Minorities”):kw | 221 |
| S6 | #2 OR #3 OR #4 OR #5 | 18,628 |
| S7 | (mHealth) OR (“m-health”) OR (“mobile health”) OR (“mobile app*”) OR (ehealth) | 8,012 |
| S8 | (“e-health”) OR (Internet) OR (“online health”) OR (“digital health”) OR (“digital intervention*”) | 20,059 |
| S9 | (tele-medicine) OR (telemedicine) OR (telehealth) OR (telecare) OR (“health technolog*”) | 13,557 |
| S10 | (“digital app*”) OR (“smartwatch”) OR (“electronic health”) OR (“Digital Health”):kw OR (“Telemedicine”):kw | 9,011 |
| S11 | #7 OR #8 OR #9 OR #10 | 39,168 |
| S12 | #1 AND #6 AND #11 | **34** |

**4. EMBASE (Elsevier)**

The search was conducted on 26^th^ May 2024.

| **Search** | **Query** | **Records retrieved** |
| --- | --- | --- |
| #1 | 'impaired glucose tolerance'/exp | 40,326 |
| #2 | 'pre-diabet*' OR prediab* OR 'pre diab*' OR 'prediabetic state' | 26,678 |
| #3 | #1 OR #2 | 47,410 |
| #4 | 'culturally and linguistically diverse' OR 'non-english' OR 'culturally diverse' OR 'linguistically diverse' OR 'low english' OR ethnic* OR migrant* OR immigrant* OR emigrant* OR 'language barrier' | 467,365 |
| #5 | 'ethnic group'/exp OR 'minority group'/exp OR 'limited english proficiency'/exp OR 'cultural diversity'/exp OR 'migrant'/exp OR 'ethnicity'/exp | 462,153 |
| #6 | #4 OR #5 | 670,372 |
| #7 | 'telehealth'/exp OR 'telemedicine'/exp OR 'digital health technology'/exp OR 'digital health'/exp | 95,649 |
| #8 | 'mhealth' OR 'm-health' OR 'mobile health' OR 'mobile app*' OR 'ehealth' OR 'e-health' OR 'internet' OR 'online health' OR 'digital health' OR 'digital intervention*' OR 'tele-medicine' OR 'telemedicine' OR 'telehealth' OR 'telecare' OR 'health technolog*' OR 'digital app*' OR 'smartwatch' OR 'electronic health' | 429,061 |
| #9 | #7 OR #8 | 448,423 |
| #10 | #3 AND #6 AND #9 | **77** |

**5. ProQuest Dissertations & theses**

The search was conducted on 26^th^ May 2024.

| **Search** | **Query** | **Records retrieved** |
| --- | --- | --- |
| S1 | summary('culturally AND linguistically diverse' OR 'non-english' OR 'culturally diverse' OR 'linguistically diverse' OR 'low english' OR ethnic* OR migrant* OR immigrant* OR emigrant* OR 'language barrier') OR diskw(Cultural diversity) OR diskw(Transients AND migrants) OR diskw(Emigrants AND Immigrants+) OR diskw(Ethnicity) OR diskw(Limited English Proficiency) OR diskw(Communication Barriers) OR diskw(Ethnic AND Racial Minorities) | 132,897 |
| S2 | summary('mhealth' OR 'm-health' OR 'mobile health' OR 'mobile app*' OR 'ehealth' OR 'e-health' OR 'internet' OR 'online health' OR 'digital health' OR 'digital intervention*' OR 'tele-medicine' OR 'telemedicine' OR 'telehealth' OR 'telecare' OR 'health technolog*' OR 'digital app*' OR 'smartwatch' OR 'electronic health') OR diskw(Digital Health) OR diskw(Telemedicine) | 147,263 |
| S3 | [summary('pre-diabet*' OR prediab* OR 'pre diab*') OR diskw('prediabetic state')](https://www.proquest.com/recentsearches.recentsearchtabview.recentsearchesgridview.scrolledrecentsearchlist.checkdbssearchlink:rerunsearch/D52D7F9039214F95PQ/None/$N?site=pqdtglobal&t:ac=RecentSearches) | 3,405 |
| S4 | [[S1] AND [S2] AND [S3]](https://www.proquest.com/recentsearches.recentsearchtabview.recentsearchesgridview.scrolledrecentsearchlist.checkdbssearchlink:rerunsearch/48C371082D194DCEPQ/None/$N?site=pqdtglobal&t:ac=RecentSearches) | 28 |
| S5 | Narrowed by:  Language:  English | **26** |

**6. ClinicalTrials.gov**

The search was conducted on 26^th^ May 2024.

| **Search** | **Query** | **Records retrieved** |
| --- | --- | --- |
| Condition/  disease | Prediabetes  Synonyms of conditions or disease (4):   Prediabetic State; Glucose Intolerance; pre-diabetes; Impaired glucose tolerance | **21** |
| Intervention/  treatment | Digital Intervention |  |

**7. ANZCTR (the Australian New Zealand Clinical Trials Registry)**

The search was conducted on 26^th^ May 2024.

| **Search** | **Query** | **Records retrieved** |
| --- | --- | --- |
| Description of intervention(s)/ exposure: | Digital | **1** |
| Study type: | Interventional |  |
| Health condition(s) or problem(s) studied: | Prediabetes |  |

**8. WHO ICTRP (the International Clinical Trials Registry Platform (ICTRP)**

The search was conducted on 26^th^ May 2024.

| **Search** | **Query** | **Records retrieved** |
| --- | --- | --- |
| 1 | (('pre-diabet*' OR prediab* OR 'pre diab*' OR 'prediabetic state') AND ('mhealth' OR 'm-health' OR 'mobile health' OR 'mobile app*' OR 'ehealth' OR 'e-health' OR 'internet' OR 'online health' OR 'digital health' OR 'digital intervention*' OR 'tele-medicine' OR 'telemedicine' OR 'telehealth' OR 'telecare' OR 'health technolog*' OR 'digital app*' OR 'smartwatch' OR 'electronic health')) | **15** |
| 2 | Recruitment status is ALL |  |

9**. Open Grey**

The search was conducted on 26^th^ May 2024.

| **Search** | **Query** | **Records retrieved** |
| --- | --- | --- |
| 1 | (('pre-diabet*' OR prediab* OR 'pre diab*' OR 'prediabetic state') AND ('mhealth' OR 'm-health' OR 'mobile health' OR 'mobile app*' OR 'ehealth' OR 'e-health' OR 'internet' OR 'online health' OR 'digital health' OR 'digital intervention*' OR 'tele-medicine' OR 'telemedicine' OR 'telehealth' OR 'telecare' OR 'health technolog*' OR 'digital app*' OR 'smartwatch' OR 'electronic health')) AND (migrant* OR immigrant* OR emigrant*) | **12** |

## Table S2. Studies excluded at full text review.

| References | Reason for exclusion |
| --- | --- |
| Abdelhameed F, Pearson E, Parsons N, et al. Health outcomes following engagement with a digital health tool among people with prediabetes and type 2 diabetes: prospective evaluation study. JMIR Diabetes [Internet]. 2023;8:e47224. Available from: <https://www.ncbi.nlm.nih.gov/pmc/articles/PMC10784975/> | Ineligible participant characteristics |
| Actrn. Evaluating Feijoa for diabetes prevention in a multi-ethnic New Zealand cohort: the FERDINAND study. A community nutrition intervention in individuals with prediabetes [Internet]. 2022. Available from: <https://trialsearch.who.int/Trial2.aspx?TrialID=ACTRN12622000210774> | Ongoing trials or study protocols |
| Dobs AS. Personalized smartphone-assisted coaching system to improve glucose homeostasis in adults with prediabetes - main study [Internet]. Available from: <https://clinicaltrials.gov/study/NCT03315663> | Ongoing trials or study protocols |
| Al-Hamdan R, Avery A, Al-Disi D, et al. Efficacy of lifestyle intervention program for Arab women with prediabetes using social media as an alternative platform of delivery. J Diabetes Investig [Internet]. 2021;12(10):1872-1880. Available from: <http://dx.doi.org/10.1111/jdi.13531> | Ineligible participant characteristics |
| Albert SL, Massar RE, Kwok L, et al. Pilot plant-based lifestyle medicine program in an urban public healthcare system: evaluating demand and implementation. Am J Lifestyle Med [Internet]. 2024;18(3):403-419. Available from: <https://journals.sagepub.com/doi/10.1177/15598276221113507> | Ineligible participant characteristics |
| Almousa Z. Pilot study of the feasibility of a worksite plant-based diabetes prevention program [Internet]. 2021:215. Available from: <https://www.sciencedirect.com/science/article/pii/S2773065423000019> | Ineligible participant characteristics |
| Ang IYH, Tan KXQ, Tan C, et al. A personalized mobile health program for type 2 diabetes during the COVID-19 pandemic: single-group pre-post study. JMIR Diabetes [Internet]. 2021;6(3):e25820. Available from: <https://www.ncbi.nlm.nih.gov/pmc/articles/PMC8274679/> | Ineligible participant characteristics |
| Anillo Arrieta LA, Flórez Lozano KC, Tuesca Molina R, et al. Glycemic status and health-related quality of life in populations at risk of diabetes in two Latin American cities. Qual Life Res [Internet]. 2023;32(8):2361-2373. Available from: <https://www.ncbi.nlm.nih.gov/pmc/articles/PMC10328894/> | Ineligible participant characteristics |
| Auster-Gussman LA, Lockwood KG, Graham SA, et al. Reach of a fully digital diabetes prevention program in health professional shortage areas. Popul Health Manag. 2022;25(4):441-448. | Ineligible participant characteristics |
| Barthold D, Chiguluri V, Gumpina R, et al. Health care utilization and medical cost outcomes from a digital diabetes prevention program in a Medicare Advantage population. Popul Health Manag [Internet]. 2020;23(6):414-421. Available from: <https://web-p-ebscohost-com.ezproxy.ecu.edu.au/ehost/detail/detail?vid=1&sid=cf3061a3-0689-49fd-a5c8-7d2fafab6d8d%40redis&bdata=JnNpdGU9ZWhvc3QtbGl2ZSZzY29wZT1zaXRl#AN=147345947&db=cul> | Ineligible participant characteristics |
| Barthow C, Hood F, McKinlay E, et al. Food 4 Health - He Oranga Kai: assessing the efficacy, acceptability and economic implications of Lactobacillus rhamnosus HN001 and β-glucan to improve glycated haemoglobin, metabolic health, and general well-being in adults with prediabetes: study protocol for a 2 × 2 factorial design, parallel group, placebo-controlled randomized controlled trial, with embedded qualitative study and economic analysis. Trials [Internet]. 2019;20(1):464. Available from: <https://www.ncbi.nlm.nih.gov/pmc/articles/PMC6664750/> | Ongoing trials or study protocols |
| Barthow C, Krebs J, McKinlay E. A multiple case study of prediabetes care undertaken by general practice in Aotearoa/New Zealand: de-incentivised and de-prioritised work. BMC Prim Care [Internet]. 2023;24(1):1-14. Available from: <https://bmcprimcare.biomedcentral.com/articles/10.1186/s12875-023-02053-1> | Ineligible study design |
| Baus A, Shawley-Brzoska S, Wright J, et al. Informatics-supported diabetes prevention programming in West Virginia. Perspect Health Inf Manag [Internet]. 2021;1-8. Available from: <https://www.ncbi.nlm.nih.gov/pmc/articles/PMC8120671/> | Ineligible participant characteristics |
| Bender MS, Cooper BA, Flowers E, et al. Filipinos Fit and Trim - a feasible and efficacious DPP-based intervention trial. Contemp Clin Trials Commun. 2018;12:76-84. | Duplicate study |
| Birse CE, McPhaul MJ, Arellano AR, et al. Impact of a digital diabetes prevention program on estimated 8-year risk of diabetes in a workforce population. J Occup Environ Med [Internet]. 2022;64(10):881-888. Available from: <https://journals.lww.com/joem/fulltext/2022/10000/impact_of_a_digital_diabetes_prevention_program_on.11.aspx> | Ineligible participant characteristics |
| Blancas-Sánchez IM, Del Rosal Jurado M, Aparicio-Martínez P, et al. A Mediterranean-diet-based nutritional intervention for children with prediabetes in a rural town: a pilot randomized controlled trial. Nutrients [Internet]. 2022;14(17):3614. Available from: <https://www.mdpi.com/2072-6643/14/17/3614> | Ineligible intervention |
| Block G, Azar KM, Block TJ, et al. A fully automated diabetes prevention program, Alive-PD: program design and randomized controlled trial protocol. JMIR Res Protoc [Internet]. 2015;4(1):e3. Available from: <http://www.ncbi.nlm.nih.gov/pubmed/25608692> | Ineligible outcomes |
| Cenčič A, Prosen M, Ličen S. Mixed-methods research on diabetes patient health education using digital technologies. Kontakt - J Nurs Soc Sci Relat Health Illn [Internet]. 2022;24(2):123-130. Available from: <https://kont.zsf.jcu.cz/pdfs/knt/2022/02/04.pdf> | Ineligible participant characteristics |
| Cha E, Kim KH, Umpierrez G, et al. A feasibility study to develop a diabetes prevention program for young adults with prediabetes by using digital platforms and a handheld device. Diabetes Educ. 2014;40(5):626-637. | Ineligible participant characteristics |
| Choi J, Lee J, Vittinghoff E, et al. mHealth physical activity intervention: a randomized pilot study in physically inactive pregnant women. Matern Child Health J [Internet]. 2016;20(5):1091-1101. Available from: <https://www.ncbi.nlm.nih.gov/pmc/articles/PMC4826820/> | Ineligible participant characteristics |
| Chong SOK, Pedron S, Abdelmalak N, Laxy M, Stephan A-J. An umbrella review of effectiveness and efficacy trials for app-based health interventions. NPJ Digit Med. 2023;6(1):1-11. | Ineligible study design |
| Coughlin SS, Heboyan V, Young L, De Leo G, Wilkins T. Use of a web portal by adult patients with pre-diabetes and type 2 diabetes mellitus seen in a family medicine outpatient clinic. J Hosp Manag Health Policy [Internet]. 2018;2. Available from: <https://www.ncbi.nlm.nih.gov/pmc/articles/PMC5976245/> | Ineligible participant characteristics |
| Coventry P, Bower P, Blakemore A, et al. Satisfaction with a digitally-enabled telephone health coaching intervention for people with non-diabetic hyperglycemia. NPJ Digit Med [Internet]. 2019;2. Available from: <https://www.ncbi.nlm.nih.gov/pmc/articles/PMC6550206/> | Ineligible participant characteristics |
| Dang S, Oropesa L, Byrne MM, et al. Race/ethnic disparities in weight and glycemia in older adults receiving lifestyle interventions via peer-leaders with or without mobile enhancement for diabetes prevention and management. J Am Geriatr Soc. 2014;62:S216. | Full-text unavailable |
| Sarfati D. BetaMe. An innovative management of diabetes and prediabetes with a comprehensive digital health programme: a randomized controlled trial. A mobile- and web-based health intervention program for diabetes and prediabetes self-management (BetaMe/Melon): process evaluation following a randomized controlled trial [Internet]. 2017. Available from: <https://www.ncbi.nlm.nih.gov/pmc/articles/PMC5836439/> | Ongoing trials or study protocols |
| Ho MM. A smartphone-based intervention for diabetes prevention in overweight Chinese adults with prediabetes [Internet]. Available from: <https://clinicaltrials.gov/study/NCT04875780> | Ineligible participant characteristics |
| Drake C, Snyderman R, Cannady M, et al. Personalized medical group visits: a novel approach for the care of prediabetes. Diabetes Spectr. 2022;35(4):504-511. | Ineligible intervention |
| Dyer KE, Moreau JL, Finley E, et al. Tailoring an evidence-based lifestyle intervention to meet the needs of women veterans with prediabetes. Women Health [Internet]. 2020;60(7):748-762. Available from: <https://www.ncbi.nlm.nih.gov/pmc/articles/PMC8435559/> | Ineligible participant characteristics |
| Edney S, Chua XH, Müller AM, Kui KY, Müller-Riemenschneider F. mHealth interventions targeting movement behaviors in Asia: a scoping review. Obes Rev [Internet]. 2022;23(4):e13396. Available from: <http://ezproxy.ecu.edu.au/login?url=https://search.ebscohost.com/login.aspx?direct=true&db=cmedm&AN=34927346&site=ehost-live&scope=site> | Ineligible study design |
| Fernández-Ruiz VE, Solé-Agustí M, Armero-Barranco D, Cauli O. Weight loss and improvement of metabolic alterations in overweight and obese children through the I2AO2 family program: a randomized controlled clinical trial. Biol Res Nurs [Internet]. 2021;23(3):488-503. Available from: <https://journals.sagepub.com/doi/10.1177/1099800420987303> | Ineligible participant characteristics |
| Fitzner K, Moss G. Telehealth—an effective delivery method for diabetes self-management education? Popul Health Manag [Internet]. 2013;16(3):169-177. Available from: <https://web-p-ebscohost-com.ezproxy.ecu.edu.au/ehost/pdfviewer/pdfviewer?vid=1&sid=7bbd0e58-18ce-4274-9594-17a34fa0af54%40redis> | Ineligible study design |
| Formagini T, Saint Onge JM, O’Brien MJ, Ramírez M, Brooks JV. The experience of Spanish-speaking Latinos in maintaining dietary and physical activity changes after the National Diabetes Prevention Program. Am J Health Educ [Internet]. 2023;54(3):224-232. Available from: <https://www.tandfonline.com/doi/full/10.1080/19325037.2023.2187487> | Ineligible study design |
| Fundació d’investigació Sanitària de les Illes Balears. mHealth intervention to prevent type 2 diabetes mellitus (Phase II study) [Internet]. Available from: <https://clinicaltrials.gov/study/NCT05110625> | Ongoing trials or study protocols |
| Gamble A, Beech BM, Wade BC, et al. Telehealth diabetes prevention intervention for the next generation of African American youth: protocol for a pilot trial. JMIR Res Protoc [Internet]. 2021;10(3):e25699. Available from: <http://ezproxy.ecu.edu.au/login?url=https://search.ebscohost.com/login.aspx?direct=true&db=cmedm&AN=33787504&site=ehost-live&scope=site> | Ineligible study design |
| Gholami M, Jackson NJ, Loeb T, et al. Twelve-month reach and effectiveness of a university-based diabetes prevention initiative. Am J Prev Med [Internet]. 2024;66(2):299-306. Available from: <http://dx.doi.org/10.1016/j.amepre.2023.09.018> | Ineligible intervention |
| Gonzalez Castro F, Shaibi GQ, Boehm-Smith E. Ecodevelopmental contexts for preventing type 2 diabetes in Latino and other racial/ethnic minority populations. J Behav Med [Internet]. 2009;32(1):89-105. Available from: <https://www.ncbi.nlm.nih.gov/pmc/articles/PMC2798813/> | Ineligible intervention |
| Grant SJ, Bensoussan A, Chang D, et al. Chinese herbal medicines for people with impaired glucose tolerance or impaired fasting blood glucose. Cochrane Database Syst Rev [Internet]. 2009;(4). Available from: <http://dx.doi.org/10.1002/14651858.CD006690.pub2> | Ineligible study design |
| Hanafiah ANM, Aagaard-Hansen J, Cheah JCH, et al. Effectiveness of a complex, pre-conception intervention to reduce the risk of diabetes by reducing adiposity in young adults in Malaysia: The Jom Mama project—a randomized controlled trial. J Glob Health [Internet]. 2022;12:1-10. Available from: <https://www.ncbi.nlm.nih.gov/pmc/articles/PMC9380849/> | Ineligible intervention |
| Heisler M, Kullgren J, Richardson C, et al. Study protocol: using peer support to aid in prevention and treatment in prediabetes (UPSTART). Contemp Clin Trials [Internet]. 2020;95. Available from: <http://dx.doi.org/10.1016/j.cct.2020.106048> | Ineligible study design |
| Ho M, Chau PH, Yu EYT, et al. Community-based weight loss programme targeting overweight Chinese adults with prediabetes: study protocol of a randomized controlled trial. BMJ Open [Internet]. 2020;10(4):e035196. Available from: <http://ezproxy.ecu.edu.au/login?url=https://search.ebscohost.com/login.aspx?direct=true&db=cmedm&AN=32273317&site=ehost-live&scope=site> | Ineligible study design |
| Hu L, Wyatt LC, Mohsin F, et al. Characterizing technology use and preferences for health communication in South Asian immigrants with prediabetes or diabetes: cross-sectional descriptive study. JMIR Form Res [Internet]. 2024;8:e52687. Available from: <https://www.ncbi.nlm.nih.gov/pmc/articles/PMC11087851/> | Full-text or data unavailable |
| Islam S, Elaiho C, Arniella G, et al. A pilot study to examine the feasibility and acceptability of a virtual adaptation of an in-person adolescent diabetes prevention program. Int J Environ Res Public Health [Internet]. 2022;19(19). Available from: <http://dx.doi.org/10.3390/ijerph191912286> | Ineligible study design |
| John T, Elsaid M, Rustgi VK. A telemedicine pilot for diabetes with applicability to NAFLD/NASH. Hepatology [Internet]. 2019;70(suppl 1):455A. Available from: <https://www.embase.com/search/results?subaction=viewrecord&id=L631815427&from=export> | Full-text unavailable |
| Jooste BR, Kolivas D, Brukner P, Moschonis G. Effectiveness of technology-enabled, low carbohydrate dietary interventions, in the prevention or treatment of type 2 diabetes mellitus in adults: a systematic literature review of randomized controlled and non-randomized trials. Nutrients [Internet]. 2023;15(20):4362. Available from: <http://ezproxy.ecu.edu.au/login?url=https://search.ebscohost.com/login.aspx?direct=true&db=cul&AN=173319418&site=ehost-live&scope=site> | Ineligible study design |
| Ma J. The PIVOT Trial: Project on EHR-integrated lifestyle interventions for adults aged fifty and older [Internet]. Available from: <https://clinicaltrials.gov/study/NCT05654142> | Ongoing trials or study protocols |
| Kaiser Permanente. Sleep for Health Study on the Effects of Cognitive Behavioral Therapy for Insomnia on Diabetes Risk [Internet]. Available from: <https://clinicaltrials.gov/study/NCT06067139> | Ongoing trials or study protocols |
| Moravcová K. Impact of digital therapeutic on metabolic parameters [Internet]. Available from: <https://clinicaltrials.gov/study/NCT04573296> | Ongoing trials or study protocols |
| Katula JA, Dressler EV, Kittel CA, et al. Effects of a digital diabetes prevention program: an RCT. Am J Prev Med [Internet]. 2022;62(4):577. Available from: <https://www.sciencedirect.com/science/article/pii/S0749379721006000?via%3Dihub> | Full-text or data unavailable |
| Khunti K, Griffin S, Brennan A, et al. Promoting physical activity in a multi-ethnic population at high risk of diabetes: the 48-month PROPELS randomized controlled trial. BMC Med. 2021;19(1):1-13. | Ineligible participant characteristics |
| Khunti K, Griffin S, Davies MJ, et al. Promoting long-term physical activity in prediabetes: the PROPELS RCT. Diabetes [Internet]. 2020;69. Available from: <https://www.cochranelibrary.com/central/doi/10.1002/central/CN-02203731/full> | Duplicate study |
| Khurshid A, Brown L, Mukherjee S, et al. Texting for health: an evaluation of a population approach to type 2 diabetes risk reduction with a personalized message. Diabetes Spectr [Internet]. 2015;28(4):268-275. Available from: <https://www.ncbi.nlm.nih.gov/pmc/articles/PMC4647170/> | Ineligible outcomes |
| Kim SE, Castro Sweet CM, Gibson E, et al. Evaluation of a digital diabetes prevention program adapted for the Medicaid population: study design and methods for a non-randomized, controlled trial. Contemp Clin Trials Commun. 2018;10:168-175. | Ineligible outcomes |
| Kirley K, Sachdev N. Digital health—supported lifestyle change programs to prevent type 2 diabetes. Diabetes Spectr [Internet]. 2018;31(4):303-309. Available from: <https://www.ncbi.nlm.nih.gov/pmc/articles/PMC6243222/> | Ineligible study design |
| Lachance L, Kelly RP, Wilkin M, et al. Community-based efforts to prevent and manage diabetes in women living in vulnerable communities. J Community Health [Internet]. 2018;43(3):508-517. Available from: <https://link.springer.com/article/10.1007/s10900-017-0444-2> | Ineligible outcomes |
| Brummelhuis LT. Preventing risk for metabolic syndrome in workaholics: an intervention [Internet]. Available from: <https://clinicaltrials.gov/study/NCT04183907> | Ongoing trials or study protocols |
| Marrero DG, Palmer KNB, Phillips EO, et al. Comparison of commercial and self-initiated weight loss programs in people with prediabetes: a randomized control trial. Am J Public Health [Internet]. 2016;106(5):949-956. Available from: <https://www.ncbi.nlm.nih.gov/pmc/articles/PMC4985082/> | Ineligible participant characteristics |
| Heisler ME. A mixed methods pilot randomized controlled trial of a mobile phone-based health program among adults with prediabetes [Internet]. Available from: <https://clinicaltrials.gov/study/NCT03025607> | Ongoing trials or study protocols |
| Matthews PH, Darbisi C, Sandmann L, et al. Disseminating health information and diabetes care for Latinos via electronic information kiosks. J Immigr Minor Health. 2009;11(6):520-526. | Ineligible outcomes |
| McAtee JR. Home food availability, food choices, and intervention strategies among prediabetes and type 2 diabetes patients [Internet]. 2019:125. Available from: <https://www.proquest.com/dissertations-theses/home-food-availability-choices-intervention/docview/2292912781/se-2?accountid=10675> | Ineligible participant characteristics |
| Millard A, Graham M, Wang X, et al. Pilot of a diabetes primary prevention program in a hard-to-reach, low-income, immigrant Hispanic population. J Immigr Minor Health [Internet]. 2011;13(5):906-913. Available from: <https://link.springer.com/article/10.1007/s10903-010-9412-y> | Ineligible outcomes |
| Moin T, Damschroder LJ, Youles B, et al. Implementation of a prediabetes identification algorithm for overweight and obese veterans. J Rehabil Res Dev [Internet]. 2016;53(6):853-862. Available from: <https://www.rehab.research.va.gov/jour/2016/536/pdf/JRRD-2015-06-0104.pdf> | Ineligible participant characteristics |
| Moin T, Martin JM, Mangione CM, et al. Choice of intensive lifestyle change and/or metformin after shared decision making for diabetes prevention: results from the Prediabetes Informed Decisions and Education (PRIDE) study. Med Decis Making [Internet]. 2021;41(5):607-613. Available from: <https://www.ncbi.nlm.nih.gov/pmc/articles/PMC8192339/> | Ineligible outcomes |
| Morris D. Interventions that prevent prediabetes from becoming type 2...second in a series. Independent Nurse. 2015:36-37. | Ineligible study design |
| Naseman KW, Faiella AS, Lambert GM. Pharmacist-provided diabetes education and management in a diverse, medically underserved population. Diabetes Spectr [Internet]. 2020;33(2):210-214. Available from: <https://www.ncbi.nlm.nih.gov/pmc/articles/PMC7228821/> | Ineligible participant characteristics |
| NCT. A mobile-based diabetes prevention program [Internet]. 2012. Available from: <https://clinicaltrials.gov/study/NCT01579292?tab=history&a=6#version-content-panel> | Ineligible outcomes |
| NCT. Community-based adolescent diabetes prevention program [Internet]. 2015. Available from: <https://clinicaltrials.gov/study/NCT02458131?tab=history&a=8#version-content-panel> | Ineligible outcomes |
| NCT. Integrating cultural aspects into diabetes education [Internet]. 2022. Available from: <https://clinicaltrials.gov/study/NCT01579292?tab=history&a=6#version-content-panel> | Ongoing trials or study protocols |
| NCT. Peer-led and telehealth comparative effectiveness research (CER) adoption for diabetes prevention and management [Internet]. 2011. Available from: <https://www.cochranelibrary.com/central/doi/10.1002/central/CN-01532259/full> | Ongoing trials or study protocols |
| NCT. Promote health with digital tools among adults with type 2 diabetes/prediabetes and/or hypertension [Internet]. 2022. Available from: <https://www.cochranelibrary.com/central/doi/10.1002/central/CN-02505826/full> | Ongoing trials or study protocols |
| Ng BP, Lamanna JB, Massey M, et al. Digital divide and Medicare Diabetes Prevention Program of beneficiaries with elevated BMI. Diabetes [Internet]. 2023;72. Available from: <http://dx.doi.org/10.2337/db23-68-OR> | Ineligible study design |
| Nguyen V, Ara P, Simmons D, Osuagwu UL. The role of digital health technology interventions in the prevention of type 2 diabetes mellitus: a systematic review. Clin Med Insights Endocrinol Diabetes [Internet]. 2024;1-11. Available from: <http://ezproxy.ecu.edu.au/login?url=https://search.ebscohost.com/login.aspx?direct=true&db=cul&AN=177391268&site=ehost-live&scope=site> | Ineligible study design |
| Nieto-Martínez R, De Oliveira-Gomes D, Gonzalez-Rivas JP, et al. Telehealth and cardiometabolic-based chronic disease: optimizing preventive care in forcibly displaced migrant populations. J Health Popul Nutr [Internet]. 2023;42(1):1-10. Available from: <https://www.ncbi.nlm.nih.gov/pmc/articles/PMC10478318/> | Ineligible study design |
| Omada Health I. Preventing diabetes with digital health and coaching [Internet]. Available from: <https://clinicaltrials.gov/study/NCT03312764> | Ongoing trials or study protocols |
| Park SH, Yao J, Chua XH, et al. Diet and physical activity as determinants of continuously measured glucose levels in persons at high risk of type 2 diabetes. Nutrients [Internet]. 2022;14(2):366. Available from: <https://www.ncbi.nlm.nih.gov/pmc/articles/PMC8781180/> | Ineligible study design |
| Patel MR, Resnicow K, Lang I, et al. Solutions to address diabetes-related financial burden and cost-related nonadherence: results from a pilot study. Health Educ Behav [Internet]. 2018;45(1):101-111. Available from: <https://www.ncbi.nlm.nih.gov/pmc/articles/PMC5908467/> | Ineligible outcomes |
| Patel MS, Polsky D, Small DS, et al. Predicting changes in glycemic control among adults with prediabetes from activity patterns collected by wearable devices. NPJ Digit Med. 2021;4(1):1-7. | Ineligible participant characteristics |
| Patel RM, Misra R, Raj S, Balasubramanyam A. Effectiveness of a group-based culturally tailored lifestyle intervention program on changes in risk factors for type 2 diabetes among Asian Indians in the United States. J Diabetes Res. 2017;2017:2751980. | Ineligible participant characteristics |
| Katzmarzyk PT. Promoting successful weight loss in primary care in Louisiana using information technology [Internet]. Available from: <https://clinicaltrials.gov/study/NCT05523375> | Ongoing trials or study protocols |
| Peña-Purcell NC, Luohua J, Ory MG, Hollingsworth R. Translating an evidence-based diabetes education approach into rural African-American communities: the “Wisdom, Power, Control” program. Diabetes Spectr [Internet]. 2015;28(2):106-115. Available from: <https://www.ncbi.nlm.nih.gov/pmc/articles/PMC4433078/> | Ineligible participant characteristics |
| Popp CJ, Hu L, Kharmats AY, et al. Effect of a personalized diet to reduce postprandial glycemic response vs a low-fat diet on weight loss in adults with abnormal glucose metabolism and obesity: a randomized clinical trial. JAMA Netw Open [Internet]. 2022;5(9):e2233760. Available from: <https://www.ncbi.nlm.nih.gov/pmc/articles/PMC9520362/> | Ineligible outcomes |
| Prasad M, Fine K, Gee A, et al. A smartphone intervention to promote time-restricted eating reduces body weight and blood pressure in adults with overweight and obesity: a pilot study. Nutrients [Internet]. 2021;13(7):2148. Available from: <https://www.ncbi.nlm.nih.gov/pmc/articles/PMC8308240/> | Ineligible participant characteristics |
| Qamar Z. Development, implementation and evaluation of an online nutrition education program for South Asians in the U.S [Internet]. 2016:118. Available from: <https://oaktrust.library.tamu.edu/bitstream/handle/1969.1/158685/QAMAR-DISSERTATION-2016.pdf?sequence=1&isAllowed=y> | Ineligible study design |
| Quan J, Lee AK, Handley MA, et al. Automated telephone self-management support for diabetes in a low-income health plan: a health care utilization and cost analysis. Popul Health Manag [Internet]. 2015;18(6):412-420. Available from: <https://www.ncbi.nlm.nih.gov/pmc/articles/PMC4688461/> | Ineligible participant characteristics |
| Ritchie ND, Turk MT. Enhancing access and impact of the Medicare Diabetes Prevention Program using telehealth: a narrative review. MHealth [Internet]. 2024;10. Available from: <http://dx.doi.org/10.21037/mhealth-23-37> | Ineligible study design |
| Salmon MK, Gordon NF, Constantinou D, et al. Comparative effectiveness of lifestyle intervention on fasting plasma glucose in normal weight versus overweight and obese adults with prediabetes. Am J Lifestyle Med [Internet]. 2022;16(3):334-341. Available from: <https://www.ncbi.nlm.nih.gov/pmc/articles/PMC9189584/> | Ineligible participant characteristics |
| Tsai SA. Microlearning application for diabetes prevention [Internet]. Available from: <https://clinicaltrials.gov/study/NCT05071820> | Ongoing trials or study protocols |
| Sapre M, Elaiho CR, Brar Prayaga R, et al. The development of a text messaging platform to enhance a youth diabetes prevention program: observational process study. JMIR Form Res [Internet]. 2024;8:e45561. Available from: <https://formative.jmir.org/2024/1/e45561> | Ineligible outcomes |
| Sarfati D, McLeod M, Stanley J, et al. BetaMe: impact of a comprehensive digital health programme on HbA1c and weight at 12 months for people with diabetes and prediabetes: study protocol for a randomised controlled trial. Trials [Internet]. 2018;19(1). Available from: <http://dx.doi.org/10.1186/s13063-018-2528-4> | Ineligible study design |
| Shaikh N, Dennis K, Brown J, et al. Protocol for the development of culturally tailored mHealth messages for a diabetes prevention program (DPP) in new immigrants in the U.S.: a pilot study. Curr Dev Nutr [Internet]. 2022;6:1158. Available from: <http://dx.doi.org/10.1093/cdn/nzac072.030> | Ineligible study design |
| Sharit J, Idrees T, Andrade AD, et al. Use of an online personal health record’s Track Health function to promote positive lifestyle behaviors in veterans with prediabetes. J Health Psychol. 2018;23(5):681-690. | Ineligible participant characteristics |
| Lee S. Diabetes prevention and prediabetes management in adults [Internet]. Available from: <https://clinicaltrials.gov/study/NCT05214209> | Ongoing trials or study protocols |
| Signal V, McLeod M, Stanley J, et al. A mobile- and web-based health intervention program for diabetes and prediabetes self-management (BetaMe/Melon): process evaluation following a randomized controlled trial. J Med Internet Res [Internet]. 2020;22(12):e19150. Available from: <https://www.ncbi.nlm.nih.gov/pmc/articles/PMC7738254/> | Duplicate study |
| Signal V, McLeod M, Stanley J, et al. A mobile- and web-based health intervention program for diabetes and prediabetes self-management (BetaMe/Melon): process evaluation following a randomized controlled trial. J Med Internet Res [Internet]. 2020;22(12). Available from: <https://www.ncbi.nlm.nih.gov/pmc/articles/PMC7738254/> | Ineligible participant characteristics |
| Soderlund PD, Stuart GW, Mueller M, et al. Feasibility of motivational interviewing and physical activity counseling sessions for improving physical activity self-management for Latina women either at risk for or diagnosed with type 2 diabetes mellitus. J Transcult Nurs [Internet]. 2019;30(5):453-460. Available from: <https://journals.sagepub.com/doi/10.1177/1043659618804614> | Ineligible outcomes |
| Soler RE, Proia K, Jackson MC, et al. Nudging to change: using behavioral economics theory to move people and their health care partners toward effective type 2 diabetes prevention. Diabetes Spectr [Internet]. 2018;31(4):310-319. Available from: <https://www.ncbi.nlm.nih.gov/pmc/articles/PMC6243226/> | Ineligible study design |
| Del Prato S. Precision medicine for preventing type 2 diabetes: a step forward PRE-MED2 [Internet]. Available from: <https://clinicaltrials.gov/ct2/show/NCT05147961> | Ongoing trials or study protocols |
| Del Prato S. Precision medicine for preventing type 2 diabetes: a step forward [Internet]. Available from: <https://clinicaltrials.gov/study/NCT05147961> | Ongoing trials or study protocols |
| Cheng S. Digital diabetes prevention program study [Internet]. Available from: <https://clinicaltrials.gov/study/NCT06137963> | Ongoing trials or study protocols |
| Dixon S. An open-label pilot intervention trial to prevent diabetes in prediabetic adult survivors of childhood cancer [Internet]. Available from: <https://clinicaltrials.gov/study/NCT04742751> | Ongoing trials or study protocols |
| Sun C, Lei Y, Lin Z, et al. Effects of self-care programs on the incidence of diabetes among adults with prediabetes: a systematic review and meta-analysis of randomized controlled trials. J Clin Nurs [Internet]. 2023;32(9-10):2193-2207. Available from: <http://ezproxy.ecu.edu.au/login?url=https://search.ebscohost.com/login.aspx?direct=true&db=cmedm&AN=35655374&site=ehost-live&scope=site> | Ineligible study design |
| Teo JYC, Ramachandran HJ, Jiang Y, et al. The characteristics and acceptance of technology-enabled diabetes prevention programs (t-DPP) amongst individuals with prediabetes: a scoping review. J Clin Nurs [Internet]. 2023;32(17-18):5562-5578. Available from: <http://ezproxy.ecu.edu.au/login?url=https://search.ebscohost.com/login.aspx?direct=true&db=cmedm&AN=36775886&site=ehost-live&scope=site> | Ineligible study design |
| Thomas NM. Caribbean (English-speaking) women in the United States: cooking for diabetes prevention and management. J Immigr Minor Health [Internet]. 2018;20(4):1025-1028. Available from: <https://link.springer.com/article/10.1007/s10903-018-0700-2> | Ineligible participant characteristics |
| Hong T. A behavioral intervention to prevent impaired glucose tolerance diabetes mellitus (DIGITAL-I) [Internet]. Available from: <https://clinicaltrials.gov/study/NCT03987438> | Ongoing trials or study protocols |
| Větrovský T. mHealth intervention to increase physical activity in prediabetes and type 2 diabetes ENERGISED [Internet]. Available from: <https://clinicaltrials.gov/show/NCT05351359> | Ongoing trials or study protocols |
| Vadheim LM, McPherson C, Kassner DR, et al. Adapted diabetes prevention program lifestyle intervention can be effectively delivered through telehealth. Diabetes Educ [Internet]. 2010;36(4):651-656. Available from: <http://dx.doi.org/10.1177/0145721710372811> | Ineligible participant characteristics |
| Ventura Marra M, Lilly CL, Nelson KR, et al. A pilot randomized controlled trial of a telenutrition weight loss intervention in middle-aged and older men with multiple risk factors for cardiovascular disease. Nutrients [Internet]. 2019;11(2):229. Available from: <https://www.ncbi.nlm.nih.gov/pmc/articles/PMC6412749/> | Ineligible participant characteristics |
| Chan WS. Digital lifestyle intervention for diabetes/prediabetes [Internet]. Available from: <https://clinicaltrials.gov/study/NCT05247437> | Ongoing trials or study protocols |
| Whitehead L, Glass C, Coppell K. The effectiveness of goal setting on glycemic control for people with type 2 diabetes and prediabetes: a systematic review and meta-analysis. J Adv Nurs [Internet]. 2022;78(5):1212-1227. Available from: <http://ezproxy.ecu.edu.au/login?url=https://search.ebscohost.com/login.aspx?direct=true&db=cul&AN=156466609&site=ehost-live&scope=site> | Ineligible study design |
| Williams A, Ford A, Webb M, et al. Public-private partnerships to lower the risk of diabetes among Black women using cooperative agreements: the National Diabetes Prevention Program and the Black Women’s Health Imperative. J Womens Health [Internet]. 2022;31(8):1079-1083. Available from: <http://ezproxy.ecu.edu.au/login?url=https://search.ebscohost.com/login.aspx?direct=true&db=cul&AN=158562776&site=ehost-live&scope=site> | Ineligible study design |
| Williams DM, Dunsiger S, Davy BM, et al. Psychosocial mediators of a theory-based resistance training maintenance intervention for prediabetic adults. Psychol Health [Internet]. 2016;31(9):1108-1124. Available from: <https://www.tandfonline.com/doi/full/10.1080/08870446.2016.1179740> | Ineligible study design |
| Pasman WJ. ELFI Health (e-Health for Empowerment by Lifestyle, Food Advice and Interaction) [Internet]. Available from: <https://clinicaltrials.gov/study/NCT06236334> | Ongoing trials or study protocols |
| Wong EM, Leung DYP, Wang Q, Leung AYM. A nurse-led lifestyle intervention using mobile application versus booklet for adults with metabolic syndrome—protocol for a randomized controlled trial. J Adv Nurs. 2020;76(1):364-372. | Ineligible study design |
| Xiong S, Lu H, Peoples N, et al. Digital health interventions for non-communicable disease management in primary health care in low-and middle-income countries. NPJ Digit Med [Internet]. 2023;6(1):1-11. Available from: <https://www.ncbi.nlm.nih.gov/pmc/articles/PMC9889958/> | Ineligible study design |
| Jeem YA. Mobile health application of screening and early intervention for prediabetic patients at primary health care settings [Internet]. Available from: <https://clinicaltrials.gov/study/NCT04979559> | Ongoing trials or study protocols |
| Manios Y. Promote health with digital tools among adults with type 2 diabetes/prediabetes and/or hypertension: DigiCare4You [Internet]. Available from: <https://clinicaltrials.gov/show/NCT05648383> | Duplicate study |
| Manios Y. Promote health with digital tools among adults with type 2 diabetes/prediabetes and/or hypertension [Internet]. Available from: <https://clinicaltrials.gov/study/NCT05648383> | Ongoing trials or study protocols |
| Zahedani AD, Veluvali A, McLaughlin T, et al. Digital health application integrating wearable data and behavioral patterns improves metabolic health. NPJ Digit Med [Internet]. 2023;6(1):1-15. Available from: <https://www.ncbi.nlm.nih.gov/pmc/articles/PMC10673832/> | Ineligible participant characteristics |
| Zhang Z, Monro J, Venn BJ. Development and evaluation of an internet-based diabetes nutrition education resource. Nutrients [Internet]. 2019;11(6):1217. Available from: <https://www.ncbi.nlm.nih.gov/pmc/articles/PMC6627433/> | Ineligible outcomes |
| Zhong Q, Chen Y, Luo M, et al. The 18-month efficacy of an intensive lifestyle modification program (ILSM) to reduce type 2 diabetes risk among rural women: a cluster randomized controlled trial. Glob Health [Internet]. 2023;19(1):6. Available from: <https://www.ncbi.nlm.nih.gov/pmc/articles/PMC9881320/> | Ineligible participant characteristics |
| Sun Z. Sports-and-medicine integrated diabetes prevention and control study: a cluster-randomized controlled trial [Internet]. Available from: <https://trialsearch.who.int/Trial2.aspx?TrialID=ChiCTR2400081848> | Full-text unavailable |
| Zou X, Luo Y, Huang Q, et al. Differential effect of interventions in patients with prediabetes: stratified by a machine learning-based diabetes progression prediction model. Diabetes Obes Metab [Internet]. 2024;26(1):97-107. Available from: <https://dom-pubs.pericles-prod.literatumonline.com/doi/10.1111/dom.15291> | Ineligible participant characteristics |
| Zheng X. Mobile internet healthcare and three disciplines co-management intervention for overweight/obese prediabetic patients [Internet]. Available from: <https://clinicaltrials.gov/ct2/show/NCT06147752> | Ongoing trials or study protocols |

## Table S3a. Quality Assessment: Randomized Controlled Trials.

| Study | Randomization used for assignment of participants to treatment groups | Allocation to treatment groups concealed | Treatment groups similar at the baseline | Participants blind to treatment assignment | Those delivering treatment blind to treatment assignment | Outcomes assessors blind to treatment assignment | Treatment groups treated identically other than the intervention of interest | Follow-up complete and if not, were differences between groups adequately described and analyzed | Participants analyzed in the groups to which they were randomized | Were outcomes measured in the same way for treatment groups | Were outcomes measured in a reliable way | Was appropriate statistical analysis used | Was the trial design appropriate, and any deviations from the standard randomized controlled trial |
| --- | --- | --- | --- | --- | --- | --- | --- | --- | --- | --- | --- | --- | --- |
| Bender [33] | Yes | Yes | Yes | No | No | Unclear | Yes | Yes | Yes | Yes | Yes | Yes | Yes |
| Block [34] | Yes | Yes | No | No | No | No | Yes | Yes | Yes | Yes | Yes | Yes | Yes |
| Fischer [35] | Yes | Yes | No | No | No | Unclear | No | Yes | Yes | Yes | Yes | Yes | Yes |
| Khunti [32] | Yes | Yes | Yes | No | No | Yes | Yes | Yes | Yes | Yes | Yes | Yes | Yes |
| Lim [36] | Yes | Yes | Yes | No | No | No | Yes | Yes | Yes | Yes | Yes | Yes | Yes |
| Marcus [37] | Yes | Yes | Yes | No | No | Unclear | Yes | Yes | Yes | Yes | Yes | Yes | Yes |
| McLeod [38] | Yes | Yes | No | No | No | Unclear | Yes | Yes | Yes | Yes | Yes | Yes | Yes |
| Nanditha [39] | Yes | Unclear | Yes | No | No | Yes | No | Yes | Yes | Yes | Yes | Yes | Yes |
| Timm [40] | Yes | Yes | Yes | No | Unclear | Unclear | Yes | Yes | Yes | Yes | Yes | Yes | Yes |
| Percentage of items assessed as met (%) | 100.0 | 88.88 | 66.66 | 0.0 | 0.0 | 22.22 | 77.77 | 100.0 | 100.0 | 100.0 | 100.0 | 100.0 | 100.0 |

Table S3b. Quality Assessment: Quasi-experimental Studies.

| Study | Clear what is the cause and what is the effect | Participants included in any comparisons similar | Participants included in any comparisons receiving similar treatment and care, other than the intervention of interest | There was a control group | Multiple measurements of the outcome both pre and post the intervention or exposure | Follow-up complete and if not, differences between groups in terms of their follow-up adequately described | Outcomes of participants included in any comparisons measured in the same way | Outcomes measured in a reliable way | Appropriate statistical analysis used |
| --- | --- | --- | --- | --- | --- | --- | --- | --- | --- |
| Collins [41] | Yes | Yes | N/A ^a^ | No | Yes | Yes | Yes | Yes | Yes |
| Kim [42] | Yes | Yes | N/A | No | Yes | N/A | Yes | Yes | Yes |
| Shin [43] | Yes | Yes | N/A | No | Yes | N/A | Yes | Yes | Yes |
| Summers [44] | Yes | Yes | N/A | No | Yes | Yes | Yes | Yes | Yes |
| Sepah [45] | Yes | Yes | N/A | No | Yes | Yes | Yes | Yes | Yes |
| Percentage of items assessed as met (%) | 100.0 | 100.0 | 0.0 | 0.0 | 100.0 | 60.0 | 100.0 | 100.0 | 100.0 |

^a^ N/A: not applicable

## Table S4. Characteristics of included studies.

| **Study** | **Country, Setting** | **Study design** | **Sample size** | **Age, range/Mean (SD)** | **Female, n%** | **Race/Ethnicity, n%** | **Language, n%** | **Year since diagnosis, mean (SD**) | | **Co-morbidities** | **Outcomes** |
| --- | --- | --- | --- | --- | --- | --- | --- | --- | --- | --- | --- |
| Lim [36] | Singapore, Primary care setting | Multicenter RCT | 148 | 21-75;  53.1 (9.3) | 39.86% | Chinese:72.3%, Malay 15.5%, Indian 0.8%, Others 0.41% | Literate in English 100% | | 2.2 (2.5) | Hypertension 81%, hyperlipidemia 81%, Others 11.5% | ITT analyses ^b^   - HbA1c - Fasting glucose - Weight loss - App utilization - Dietary intake and physical activity |
| Bender [33] | US, In the San Francisco Bay Area | RCT | 67 | 41.7 (12.0) | 52.20% | Native-born  Filipinos 46.3%, Immigrants (≤5 years in the US) 54% | English (speak and read) 100% | | N/A | BMI for Asians>23kg/m2; Hypertension | ITT analyses   - HbA1c - Fasting glucose - Weight loss - Waist circumference - BMI |
| Block [34] | US, community | RCT | 339 | 31-70;  55 (8.9) | 31.3% | White 67.6%, Hispanic6.2%, Asian20.6%, Other 5.6% | Spoke English | | N/A | BMI ≥27 kg/m2 (Asian >25 kg/m2) | ITT analyses   - HbA1c - Fasting glucose - Weight |
| McLeod [38] | New Zealand, primary care setting | Parallel-group two-arm single-blinded RCT | 429 | 18-75; 62.1 (9.06) | 50.8% | Māori 15.2%, Pacific 3.7%, all others 81.1% | Prediabetes-range group: intervention110 (51.2%), control115 (53.7%). | | N/A | Heart disease15.9%, Hypertension50.1%, Depression/anxiety24.2%, Arthritis28.7%, Lung disease14.5%, Cancer8.4%, and Chronic pain15.2% | ITT analyses   - HbA1c - Weight - BMI - Waist circumference - Systolic and diastolic blood pressure |
| Marcus [37] | US, Community | Two-arm open-labeled RCT | 199 | 18-65;  43.8 (10.11) | 100% | Mexican/Chicana 89.0%, Puerto Rican 0.5%, Cuban 0.5%, Colombian 1.0%, Other 9.6% | Speak only Spanish or more Spanish than English at home 83.4% | | N/A | N/A | ITT analyses   - HbA1c - Weight - MVPA ^a^ - BMI - Adverse events |
| Khunti [32] | UK， Primary care, and the community | Three-arm, parallel-group, pragmatic, superiority RCT with embedded qualitative sub-studies | 1366  Pre-diabetes=527; Overall prediabetes 38.58% | 40-74; median 61 years | 49% | White European  71.9%, Black and Minority Ethnic groups 28.0% | Ability to speak English 100% | | < 5 years | Median BMI28.4 kg/m², CVD 9.1% | - HbA1c - Weight - MVPA ^a^ - Waist circumference - BMI |
| Fischer [35] | US, Primary Care Setting | RCT | 163 | 46.5 (11.6) | 73% | N/A | Spanish 54.0%, English 44.2% | | N/A | Overweight/obesity 100% | ITT analyses   - HbA1c - Weight - 5% weight loss - Systolic blood pressure - Operating costs per participant |
| Nanditha [39] | India and the UK, multi-national, multi-sector, community and primary care settings | RCT | 2062 | India:35-55  UK:40-74  Overall: 52.0 (10.3) | 36% | N/A | In India, messages were in English and in two local languages. | | N/A | N/A | ITT analyses   - HbA1c - Blood pressure - BMI - Waist circumference - Fasting glucose - Lipid levels |
| Kim [42] | US, community | A single-group feasibility study | 50 | 59.3 (7.2) | 50% | Korean 100% | Poor/Very Poor in English 72%;  Always needing for translator 72%. | | 2.4 (1.2) | High blood pressure 36%, High cholesterol 36% | - HbA1c - Weight - Blood pressure - Total Cholesterol - Diabetes-related Efficacy and Knowledge - Depression - Feasibility |
| Shin [43] | US, community | A single-group, pre-posttest design | 43; Prediabetes: 18.6% | 35-64;  48 (6.9) | 69.8% | Korean 100% | Korean speaking 100% | | N/A | N/A | - HbA1c - BMI - Blood pressure - Mean Daily Steps - Self-efficacy - Behavioral Risk Factor - Social Support - Feasibility |
| Collins [41] | US, community | A pre-posttest design | 2390 | 18-88;  54.9 (10.2) | 89.2% | African American, American Indian, Asian, Hawaiian/Pacific Islander, and White | N/A | | N/A | N/A | - Self-report HbA1c - Weight - MVPA - Progress on Mindfulness Strategies - Confidence in 18 Behaviors - Adoption of 21 Behaviors |
| Summers [44] | UK, primary care setting | RCT | 27 | 52.4 (13.4) | 56% | White 89%,  Indian/Pakistan/Bangladeshi/Arabic 11% | N/A | | N/A | N/A | ITT analyses   - HbA1c - Weight - Weight loss |
| Timm [40] | Sweden， South Africa, Uganda; primary care and community settings | Pragmatic cluster RCT | Sweden: n=110  South Africa=  285 Uganda=377 | 30 -75  South Africa: 51.6 (10.3)  Uganda: 51.7  (11.3) | N/A | N/A | Swedish or English | | N/A | N/A | - HbA1c - FPG(Uganda & SA） - Weight - Waist circumference - Process evaluation - Treatment satisfaction - Out-of-pocket expenditure |
| Sepah [45] | US, N/A | Pre–post tests | 220 | 43.6 (12.4) | 62% | Caucasian 50.2%,  Black 29.3%,  Hispannic 10.7%, and other 9.8% | N/A | | <1 year | N/A | - HbA1C - Weight - Program Engagement |

^a^ Minutes-per-week of moderate-to-vigorous physical activity.

^b^ Intention-to-treat analyses.

## Table S5. Reach, uptake, engagement and feasibility outcomes of digital health interventions across studies.

| **Author, year** | **Intervention** | **Mode of delivery** | **Reach** | **Uptake** | **Engagement** | **Feasibility** |
| --- | --- | --- | --- | --- | --- | --- |
| Block et al. (2015) [37] | Alive-PD Web-based program (12 months): 6 months weekly + 6 months biweekly contact | - Web-based - Telephone calls - SMS ^a^ - Email - Mobile app | NR ^b^ | NR | - Early engagement (≥4 weeks): 87.1% - Overall participation (≥17 weeks):70.8% - Sustained engagement (at 6 months): 71.1% | - Retention rates: 3-month follow-up: 89.1%,6-month follow-up: 86.1% - Attrition rate: Intervention 15.9%, Control 11.8% |
| Bender et al.,2018 [38] | Fit&Trim program (6months):3-month intervention + 3-month maintenance | - Face-to-face - Mobile technology (Fitbit Zip, mobile app) - Social media | NR | 78.8% | - 91% attended ≥4 of 6 intervention office visits | - Retention rates: 6-month intervention:90%, 6-month follow-up 91% - Attrition rate: Intervention 15.2%, Control2.9% |
| Fischer et al. (2016) [39] | Message-augmented intervention (12 months) | - SMS - Telephone - Software | 14.6% | NR | NR | - Retention rate:96.3% - Overall attrition rate: 3.7% - Contamination: Intervention vs. Control   a) DPP ^C^ Classes: 10 vs.9 participants  b) Weight loss program: 29.2% vs. 35.6%  c) Weight loss pill: 0 vs.8.3%  d) Nutritional supplement: 8.2% vs.11.1% |
| Lim et al. (2021) [40] | An app-based lifestyle program (6 months): Nutritionist Buddy Diabetes app with in-app dietitian coaching | - Face-to-face - Mobile app | 76.4% | 100% | - App utilization:3 months: 97.8%, 4-6 Months: 91.7% - Dietitian interaction for 3 months: average 3 days/week; 4-6 Months: average 2 days/week | - Retention rates:3-month: 95.9%, 6-month: 93.2% - Attrition rates:5.4%   Intervention: 6.9%, Control: 3.9% |
| Marcus et al.,2022 [41] | Enhanced PA ^d^ Intervention: 6-month intervention + 12-month follow-up | - Face-to-face - SMS - Printed materials - Telephone calls | 21.4% | 100% | - One-week call completion: 89% - Enhanced Intervention participants:2-month: 60%,3-month: 50% | - Retention rates: 6-month follow-up: 68.6%, 12-month follow-up: 62.7% - Attrition rates:6-month: 23%,12-month: 26.7% |
| McLeod et al.,2020 [42] | BetaMe/Melon digital program (12 months): 16 weeks core program + 36 weeks maintenance | - Mobile devices - Web-based platforms | 17.6% | 75.9% | - Initial health coaching session: 92% - Online engagement (4-month): 74% overall; lower for Māori participants (40% reducing to 3%) - Diary completion: 40% reducing to 20% - Support forum: 20% reduced to 5% | - Retention rates: 4-month: 97.7% intervention, 99.5% control; 12-month: 94.4% intervention, 97.2% control - Attrition rates: 4-month: 2.3% intervention, 0.5% control; 12-month: 5.6% intervention, 2.8% control - No adverse events reported |
| Nanditha et al. (2020) [35] | SMS-based lifestyle support (24 months) | - SMS | 12.3% | NR | - Message frequency: 2-3 times/week - 75-80 tailored messages per Transtheoretical Model stage | - Acceptability: India: median score 3/6, UK: 85% at 6 months, 82% at 24 months - Attrition rates:14.5%   Intervention:149 lost, Control:150 lost   - Message disturbance: <5% of Indian participants reported messages as disturbing |
| Timm et al. (2021) [43] | SMART2D project (12 months): a telephone-facilitated health coaching intervention + community intervention | - Face-to-face (CHW) ^b^ - SMS - Telephone - A linked peer support system | - Uganda:2.8% - South Africa:35.7% - Sweden:6.7% | NR | NR | - Retention rates: Uganda (57.6%): Intervention 86.5%, Control1 61.1%, Usual care 24.8% - South Africa (49.8%): Intervention 51.0%, Control 6.4% - Sweden:1. Delivery challenges: a) Language skills barriers; b) Different delivery styles;   c) Variable goal-setting approaches. 2. Implementation factors: a) Facilitator-participant interaction is important; b) Need for intervention tailoring; c) Importance of language-skilled facilitators |
| Khunti et al. (2021) [34] | Walking Away Plus: 3-hour group session + mHealth support (48-month follow-up) | - SMS - Telephone calls | 11.9% | 87.4% | - Group session attendance: 80% - Text messaging registration (Walking Away Plus): 78% - Program adherence at 48 months (Walking Away Plus vs Walking Away vs Control):   Pedometer use: 64.2% vs 49.7% vs 19.7%  Exercise log keeping: 40.9% vs 30.6% vs 11.1%  Goal setting: 78.8% vs 73.0% vs 64.0% | - 48-month retention rate (73.6%): Control 77.1%, Walking Away 71.3%, Walking Away Plus: 71.6% - Qualitative findings: - Positive engagement reported - Tailored mHealth support considered helpful - Age-related health issues affected long-term sustainability |
| Collins et al. (2023) [44] | DPP program (12 months): 6-month core + 6-month core maintenance | - SMS - Online platforms | NR | NR | - Behavior adoption:16.6%-98.4% - Most adopted (>95%): mindful eating, reduced caloric beverages, PA awareness - Least adopted (<60%): strength training, family screen time, extended PA (60-90 min) | - Retention rates: Phase 1: 74.0%; Overall (attending ≥9/18 Phase 1 and ≥5/8 Phase 2):46.9% - Attrition rates: Phase 1: 26%, Overall: 53.1% |
| Kim et al.,2019 [36] | Self-help intervention program (hSHIP)  (6months): mobile SHIP + personal coaching | - Face-to-face - SMS - Mobile - Web | 79.4% | 100% | - Counseling session attendance rate: 96.5% - Counseling sessions completed: 630 sessions | - Retention rate:87% - Attrition rate:13.0% - Acceptability: Well-accepted by participants and community health workers |
| Shin et al. (2022) [45] | A Technology-Enhanced PA Intervention (12-week) | - Face-to-face (education sessions, group walking) - SMS - Wearable devices (Fitbit) | NR | NR | - Adherence (Fitbit wear): 93% | - Retention rates:100% - Satisfaction rate: 88.4% |
| Summers et al. (2021) [46] | Low Carb Program (12 months): 12-week core course + support for 12 months | - Digital platform accessible via: - Internet (web, app) - Mobile devices - Smart devices (watches, speakers, assistants) | 45% | 100% | - 100% completed ≥40% of lessons - 66.7% completed ≥75% core lessons - 30-day engagement: 77.8% | - Retention rates: 6-month follow-up: 77.8%, 12-month follow-up: 77.8% - Attrition rate:12-month dropout: 22.2% |
| Sepah et al. (2014) [47] | DPP-based group lifestyle intervention: 16-week core + post-core maintenance to 12 months | - A digital pedometer - SMS - Telephone calls - Online platforms - Social media | 86.6% | 100% | - Average lessons completed: 13.8 lessons - 85% completed ≥4 lessons - 65.5% completed ≥4 core + ≥1 post-core lessons | - Retention rates: Core phase:85%, post-core phase: 65.5% - Attrition rates: Core phase: 15%, Overall: 34.5% - Higher program completion was associated with being female, older age (45.3 vs 40.3 years), and higher education level (*P* <.01) |

^a^ SMS: Short Message Service; ^b^ NR: Not Report; ^C^ DPP: Diabetes Prevention Program; ^d^ PA: physical activity. Note: Reach = eligible participants/invited participants; Uptake = randomized participants/eligible participants

## Table S6. Summary of cultural adaptation strategies used in digital health interventions (4 studies).

| Author, year | Population | Study design | Digital modality | Cultural adaptation strategy | Key intervention components | Observed effects |
| --- | --- | --- | --- | --- | --- | --- |
| Shin et al.,2022 [45] | First-generation Korean Americans | A single-group, pre-posttest design | Wearable devices  + Text messages | - Community-based recruitment at Korean cultural venues (grocery stores, churches) - Small group sessions (2-3 participants) suitable for Korean American cultural preferences - Korean language delivery | • Bi-weekly face-to-face education  • Weekly reminder text messages  • Monthly group walking sessions  • Fitbit self-monitoring and goal setting | Significant improvements in self-efficacy, moderate PA ^a^, and daily steps; non-significant HbA1c change |
| Marcus et al., 2022 [41] | Latinas | Two-arm open-labeled RCT | Text messages + Telephone calls | - "Seamos Activas" - culturally targeted program - Spanish language support - SCT ^b^ adapted for Latino culture - Individual reports mapping PA locations near participants' homes | • Daily text messages with SCT ^b^-based PA ^a^ tips  • Goal-setting and PA ^a^ monitoring  • Individually tailored PA ^a^ reports and tips sheets  • Additional telephone calls for support and troubleshooting | Enhanced intervention vs controls: higher PA guideline adherence (57% vs 44%); borderline significant HbA1c reduction; no between-group MVPA ^c^ differences; both groups showed significant within-group PA ^a^ improvements |
| Kim et al.,,2019 [36] | Korean Americans | Single-group feasibility study | Text messages + Digital counseling  + Web app | - Korean language delivery - Bilingual Korean CHWs ^d^ - Culturally adapted psycho-behavioral education - Addressing Korean Americans' unique barriers (e.g., limited Korean-speaking providers, social isolation) | - Goal setting, skills training, home monitoring - Personalized text messages in Korean - Home glucose monitoring - Digital counseling with personalization and tailoring - Persuasive technology principles integrated into web app | No diabetes progression in prediabetes participants (0/50); non-significant improvements in weight, diabetes-related efficacy; blood pressure improvements at 3 months not maintained |
| Bender et al., 2018 [38] | Native-born  Filipino Americans | A pilot RCT | Fitbit Zip activity tracker  + Mobile app/ diary  + Private Facebook group | - Filipino research staff - Family involvement in sessions - Teaching healthier versions of Filipino dishes - A Filipino food guide with nutritional information - Strategies for healthy eating at Filipino family gatherings - Indoor/outdoor activities suitable for Filipino cultural preferences - Community stakeholder input through focus groups - Addressing cultural norms and barriers | - DPP-based lifestyle intervention - Goal setting for weight, diet, and PA - In-person office visits - Fitbit and Facebook for behavior tracking and virtual support - Weekly health behavior coaching | Significant reductions in weight, BMI, and waist circumference; 41% of participants achieved ≥5% weight loss; non-significant fasting glucose and HbA1c changes |

^a^ Physical activity

^b^ Social Cognitive Theory
^c^ Moderate-to-Vigorous Physical Activity

^d^ Community Health Workers
