# Supplementary material for: Effectiveness, Reach, Uptake, and Feasibility of Digital Health Interventions for Culturally and Linguistically Diverse Populations Living With Prediabetes Across the Lifespan: Systematic Review and Meta-Analysis
Source: JMIR Diabetes. 2026 Feb 19;11:e70912. doi: 10.2196/70912 (PMC12919907; doi:10.2196/70912)

**Multimedia Appendix 2**

Figure S1. Leave-one-out sensitivity analyses of HbA1c (%) showing the influence of each study on the pooled effect estimates.

Figure S2. Forest plots after removing influential studies in RCTs (Nanditha et al., 2020) and Pre-Post studies (Kim et al.,2019).

Figure S3. Meta-analysis of RCTs assessing the effect of DHIs on FPG among CaLD populations living with prediabetes.


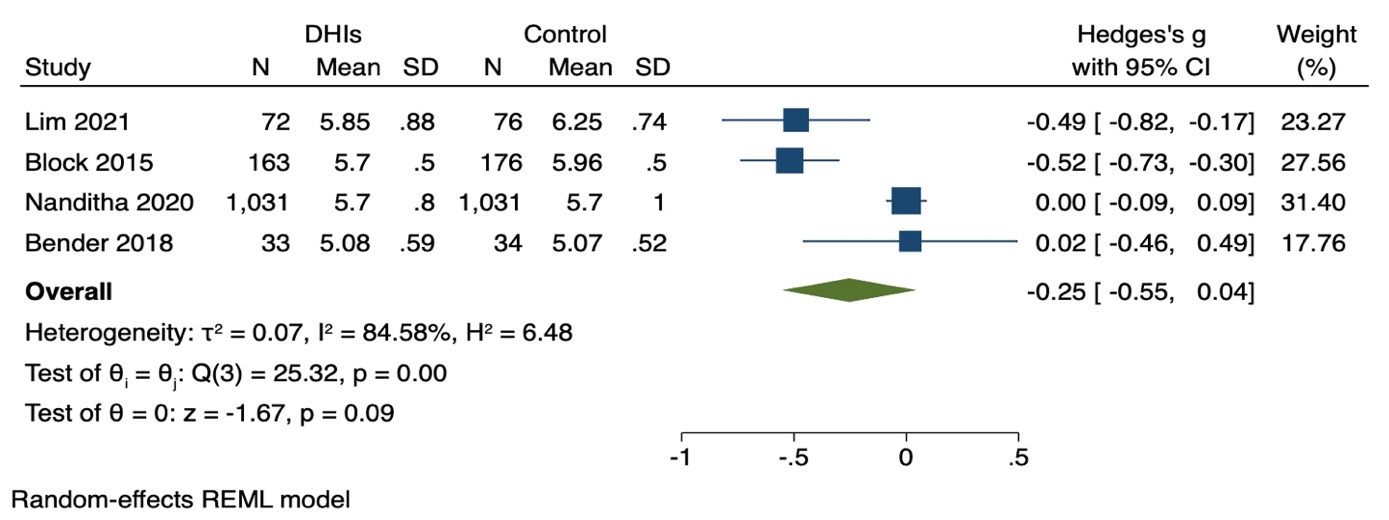


Meta-analysis of pre-post studies assessing the effect of DHIs on body weight among CaLD populations living with prediabetes.


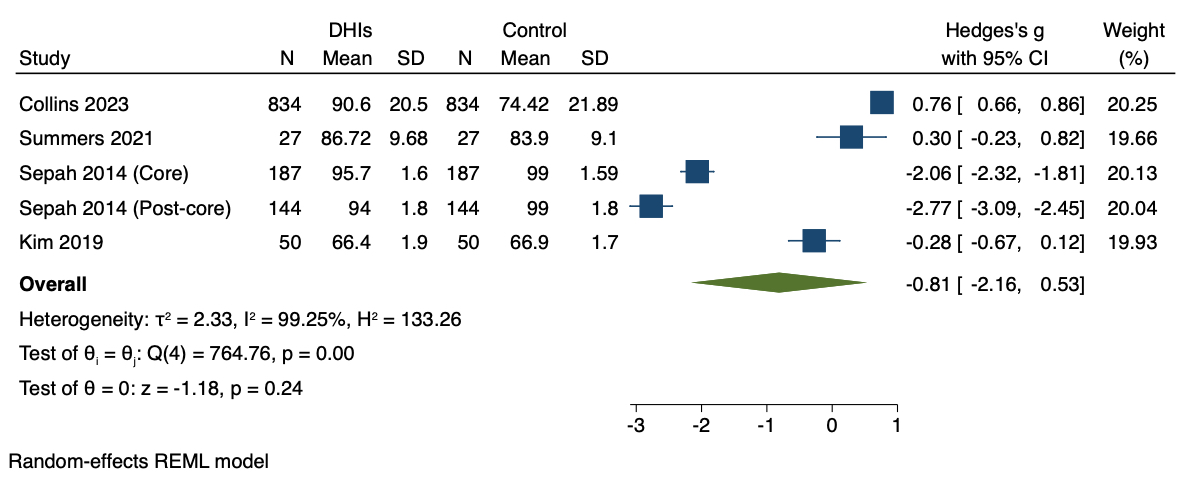


Meta-analysis of RCTs assessing the effect of DHIs on body weight among CaLD populations living with prediabetes.


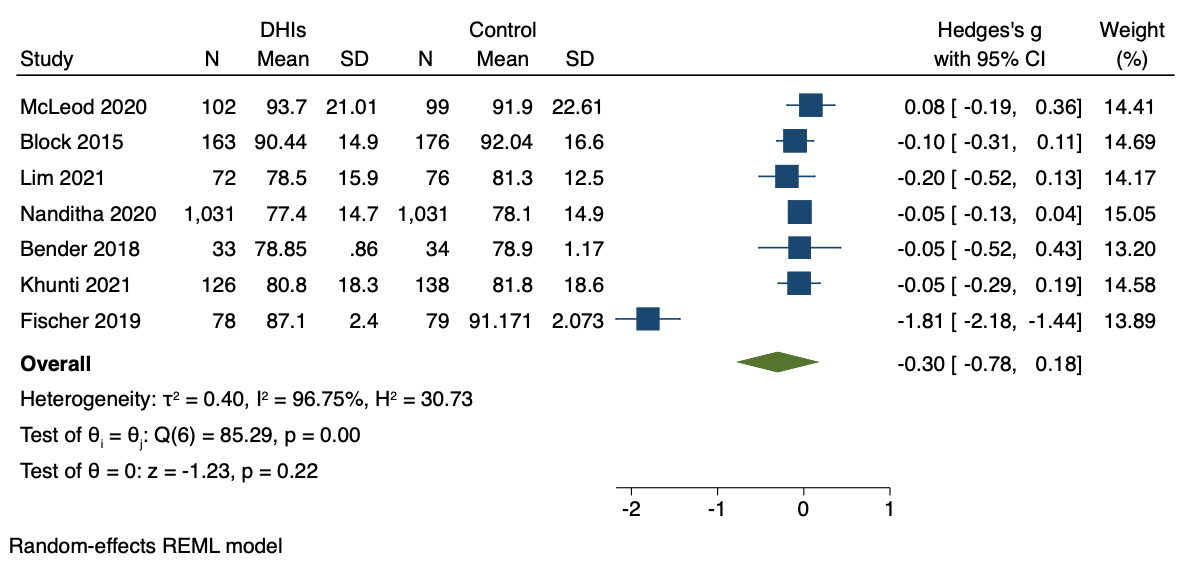

Supplement: Multimedia Appendix 2 [file diabetes-v11-e70912-s002.docx]
